# Optimizing Surface ^*^H Coverage over Cu_2_O/Co_3_O_4_ Heterojunction Enables Efficient Neutral Electrocatalytic Hydrogenation of 5‐hydroxymethylfurfural to 2,5‐dihydroxymethylfuran

**DOI:** 10.1002/advs.202513460

**Published:** 2025-09-26

**Authors:** Yun Ge, Wei Wang, Xiao‐Qiang Pan, Jia‐Wei Huang, Jie‐Jie Chen, Wu‐Jun Liu, Yuqin Zou, Han‐Qing Yu

**Affiliations:** ^1^ State Key Laboratory of Advanced Environmental Technology Department of Environmental Science and Engineering University of Science and Technology of China Hefei 230026 P. R. China; ^2^ State Key Laboratory of Chemo/Bio‐Sensing and Chemometrics, College of Chemistry and Chemical Engineering Advanced Catalytic Engineering Research Center of the Ministry of Education Hunan University Changsha Hunan 410082 P. R. China

**Keywords:** ^*^H coverage, Cu_2_O/Co_3_O_4_ heterojunction, neutral electrocatalytic hydrogenation, reaction pathway modulation

## Abstract

Electrochemical hydrogenation (ECH) of biomass‐derived 5‐hydroxymethylfurfural (HMF) to 2,5‐dihydroxymethylfuran (DHMF) offers a sustainable route for biomass valorization. Recent studies have underscored the importance of reactive hydrogen species (^*^H) on the catalyst surface in determining reaction selectivity, particularly under neutral conditions. However, the mechanistic understanding of how ^*^H coverage governs the reaction pathway remains poorly understood, and effective strategies for optimizing surface ^*^H coverage are still lacking. Herein, density functional theory (DFT) calculations first reveal that an optimum ^*^H coverage on the Cu_2_O surface effectively suppresses ketyl intermediate coupling and thermodynamically favors DHMF formation. Inspired by this insight, a Cu_2_O/Co_3_O_4_ heterojunction catalyst is constructed, in which Co_3_O_4_ serves as a redox‐active cocatalyst to stabilize Cu⁺ sites and modulate the electronic structure of Cu_2_O, thereby enhancing H_2_O activation and enabling precise tuning of ^*^H coverage. The Cu_2_O/Co_3_O_4_ heterojunction catalyst delivers an excellent HMF conversion (97%) and DHMF selectivity (97%), significantly outperforming the pristine Cu_2_O (71% DHMF selectivity, 62% HMF conversion). This work uncovers the mechanistic role of ^*^H coverage in pathway regulation and highlights heterointerface engineering as a powerful strategy for designing efficient electrocatalysts for selective biomass upgrading under neutral conditions.

## Graphical abstract

This work presents a heterojunction strategy, wherein Co_3_O_4_ functions as a redox‐active cocatalyst to stabilize Cu⁺ sites and finely tune the electronic structure of Cu_2_O. Such interfacial modulation accelerates H_2_O dissociation kinetics under neutral conditions and enables optimal surface ^*^H coverage, thereby facilitating efficient HMF electro‐hydrogenation with high selectivity toward DHMF.

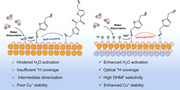

## 1 Introduction

**Figure 1:**
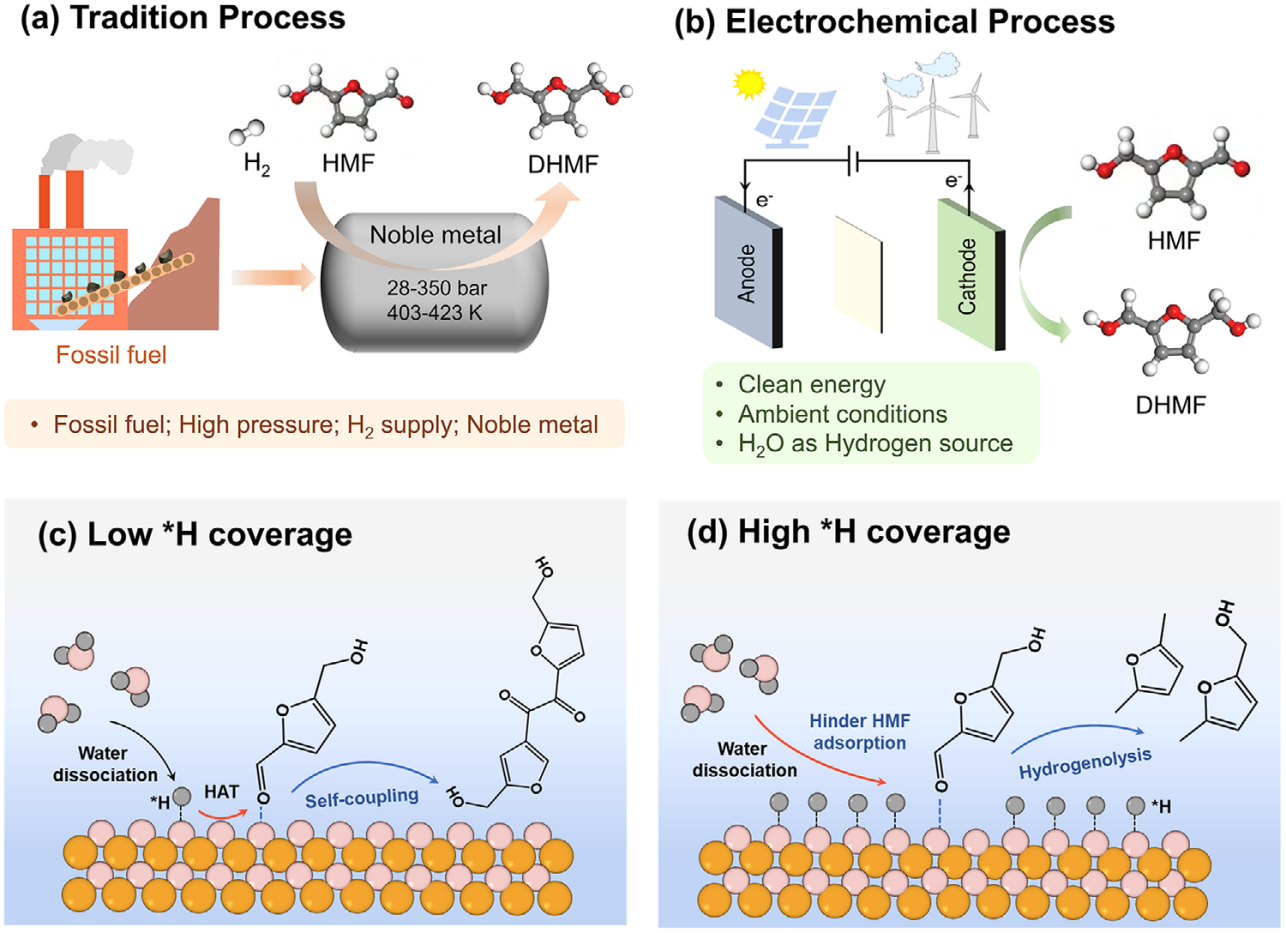
Schematic illustration comparing the electrochemical synthesis with the conventional thermocatalytic route. Comparison of the traditional thermal catalytic process a) and the electrochemical approach b) for DHMF production. Effect of low c) and high d) surface ^*^H coverage on HMF‐to‐DHMF reaction pathways. Red arrows indicate the dominant DHMF route; blue arrows show competing pathways (BHH formation and hydrogenolysis). Gold, pink, and gray balls represent Cu, O, and H atoms, respectively.

5‐Hydroxymethylfurfural (HMF), a pivotal biomass‐derived platform compound, serves as a critical bridge between the upstream biomass and downstream high‐value chemicals. Recognized by the US Department of Energy as one of the “top ten biobased chemicals,” HMF has garnered significant attention for its versatility.^[^ ^1^, ^2^ ^]^ The presence of reactive C2‐position aldehyde groups and C5‐position hydroxymethyl groups renders 5‐HMF amenable to a plethora of reactions, including hydrogenation, deoxygenation, esterification, oxidation, and condensation. Among these, the hydrogenation of 5‐HMF has been extensively investigated, given its high‐value hydrogenated products such as 2,5‐dihydroxymethylfuran (DHMF) and 2,5‐dimethylfuran (DMF). Of particular significance is DHMF, a vital precursor for producing resins, pharmaceuticals, artificial fibers, and functional polymers.^[^ ^3^, ^4^ ^]^ Currently, DHMF is predominantly produced via hydrogenation of HMF using H_2_ as a primary hydrogen source under high temperature and pressure (**Figure** **1**a).^[^ ^3^, ^5^ ^]^Although these methods have been well developed, they inevitably cause several concerns, such as high energy consumption, catalyst inactivation, safety, and sustainability.

Electrochemical hydrogenation (ECH) of HMF presents a promising alternative approach to conventional heterogeneous catalytic hydrogenation, as it proceeds under ambient conditions and eliminates the requirements of high‐pressure molecular hydrogen (Figure 1b).^[^ ^6^, ^7^ ^]^ Recent research on the ECH of HMF has developed a series of noble metal (e.g., Ag, Ru, Rh, and Pd)‐based electrocatalysts with high catalytic activity and stability,^[^ ^8^, ^9^, ^10^ ^]^ but their scarcity and high costs motivate researchers to seek abundant and inexpensive alternative catalysts. As an alternative, Cu_2_O is particularly attractive due to its favorable binding affinity toward the carbonyl group and moderate hydrogen adsorption energy.^[^ ^11^, ^12^, ^13^ ^]^ The ECH of HMF over the Cu_2_O based catalysts generally proceeds via a hydrogen atom transfer (HAT) mechanism, in which the final hydrogenation step from ketyl intermediates to DHMF highly depends on the local reactive hydrogen species (^*^H) availability of the electrodes (Figure S1, Supporting Information).^[^ ^14^, ^15^ ^]^ Generally, Insufficient ^*^H availability leads to the coupling of two ketyl intermediates to form undesired dimers, such as 5,5′‐bis(hydroxymethyl)hydrofuroin (BHH) (Figure 1c),^[^ ^8^, ^16^ ^]^ while excessive ^*^H may block active sites for HMF adsorption^[^ ^15^, ^17^ ^]^ or trigger side reactions such as hydrogenolysis^[^ ^18^ ^]^ (Figure 1d), ultimately decreasing the selectivity toward DHMF. Therefore, the reaction pathway for the ECH of HMF is strongly governed by the surface ^*^H coverage of the electrocatalysts. Despite its critical roles, the optimal range of surface ^*^H coverage on the Cu_2_O‐based catalysts remains poorly understood, and rational approaches for optimizing the surface ^*^H coverage remain underdeveloped. On the other hand, under the ECH conditions, the Cu(I) sites in the Cu_2_O tend to experience a dynamic structural reconstruction, which may result in the loss of active Cu(I) centers, thereby diminishing its catalytic activity and complicating the mechanistic understanding.^[^ ^19^, ^20^, ^21^, ^22^ ^]^ Stabilizing Cu(I) species is therefore critical to unlocking the full potential of Cu_2_O‐based electrocatalysts for the ECH of HMF, but remains a persistent challenge.

To address these challenges, one promising strategy is integrating a redox‐active cocatalyst that not only stabilizes the Cu(I) species through preferential reduction,^[^ ^22^, ^23^, ^24^ ^]^ but also modulates the electronic structure of Cu_2_O to enhance its H_2_O dissociation kinetics.^[^ ^24^, ^25^ ^]^ Based on this rationale and our previous experimental findings,^[^ ^25^, ^26^ ^]^ we identify Co_3_O_4_ as a suitable cocatalyst to form a heterojunction structure with Cu_2_O due to its redox flexibility and favorable interfacial properties. Such a heterojunction structure may offer an efficient approach to stabilize the Cu(I) active sites and modulate the surface ^*^H coverage of Cu_2_O, thus steering the reaction pathway toward DHMF formation under neutral conditions.

Herein, the density functional theory (DFT) calculations first reveal that optimum surface ^*^H coverage on Cu_2_O is crucial for promoting DHMF formation while suppressing the dimerization of two ketyl intermediates. Guided by the DFT results, we design a Cu_2_O/Co_3_O_4_ heterojunction catalyst, where Co_3_O_4_ serves as a redox‐active cocatalyst to stabilize the active Cu(I) species and enhance H_2_O dissociation by modulating the electronic structure of Cu_2_O. This regulation enables precise control of ^*^H coverage, steering the pathway for the ECH of HMF to DHMF. The as‐designed Cu_2_O/Co_3_O_4_ heterojunction catalyst delivers 97% HMF conversion and 97% DHMF selectivity under neutral conditions, significantly outperforming pristine Cu_2_O. This work demonstrates the feasibility of heterointerface engineering as a powerful approach to regulate ^*^H coverage and stabilize active centers for highly selective biomass electro‐hydrogenation.

## 2 Results and Discussion

**Figure 2:**
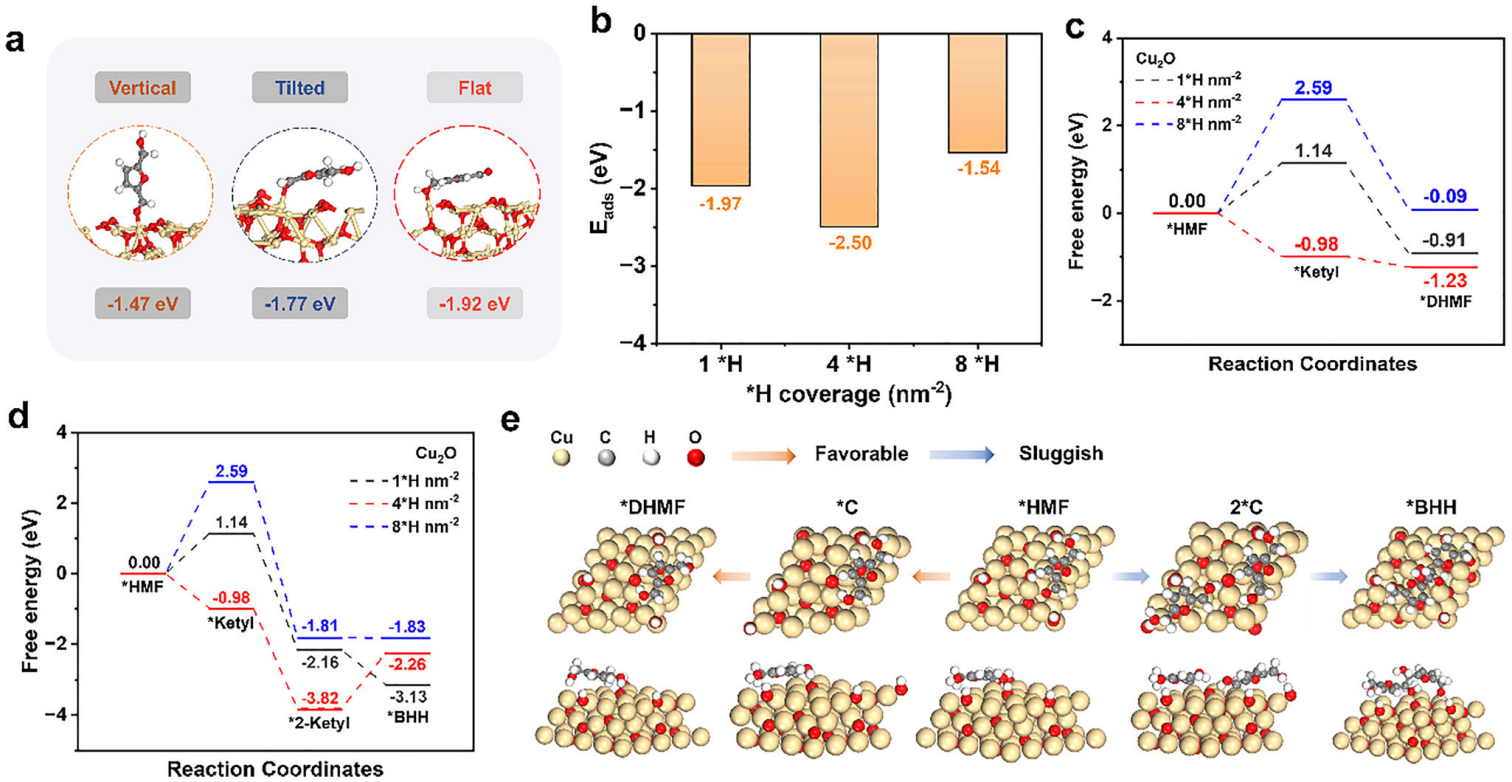
a) The adsorption configuration of HMF on Cu_2_O. b) The adsorption energy of HMF on Cu_2_O under different hydrogen coverage. Calculated free energy profiles for HMF hydrogenation to c) DHMF and d) BHH at Cu_2_O using the HAT reaction mechanism under different hydrogen coverage. e) Schematic illustration of the formation of DHMF and BHH during the ECH of HMF process on Cu_2_O under 4 ^*^H coverage.

Prior to the rational design of efficient electrocatalysts for HMF electro‐hydrogenation, it is essential to elucidate how surface ^*^H coverage governs the reaction pathway. To this end, we perform DFT calculations on Cu_2_O (111) surfaces with varying ^*^H coverages, including 1^*^H nm^−2^ (low), 4^*^H nm^−2^ (moderate), and 8^*^H nm^−2^ (high) per unit supercell (Figure S2, Supporting Information) to assess their impact on HMF adsorption and selectivity toward DHMF. Adsorption energy analysis reveals that HMF preferentially adopts a flat‐lying configuration (−3.12 eV), which is energetically more favorable than tilted (−2.26 eV) or upright (−1.98 eV) geometries (**Figure** **2**a; Figure S3, Supporting Information). Based on this configuration, we further examined HMF adsorption energies under different ^*^H coverages. As shown in Figure 2b and Figure S4 (Supporting Information), high ^*^H coverage (8 ^*^H nm^−2^) significantly weakens adsorption (−1.54 eV), likely due to site blocking and steric hindrance. In contrast, moderate coverage (4^*^H) yields the strongest adsorption (−2.50 eV), compared to −1.97 eV under low coverage (1^*^H), indicating that 4^*^H provides a favorable balance between hydrogen availability and active site accessibility.

To further clarify the effect of surface ^*^H coverage on the reaction pathway, we separately evaluated the thermodynamics of HMF conversion to DHMF and the competing dimerization to 5,5′‐bis(hydroxymethyl)hydrofuroin (BHH) at 1^*^H, 4^*^H, and 8^*^H coverage levels. For the hydrogenation pathway (HMF → carbon radical intermediate (^*^C) → DHMF), the free‐energy barrier for ^*^C formation at 1 ^*^H nm^−2^ coverage is 2.59 eV, identifying ^*^C formation as the rate‐determining step and accounting for the slow overall kinetics. Although increasing ^*^H coverage to 8^*^H reduces this barrier to 1.14 eV, the subsequent step toward DHMF remains thermodynamically uphill, compromising overall efficiency. In contrast, 4^*^H coverage renders both steps downhill in free energy, suggesting that a moderate ^*^H level provides an optimal microenvironment for stepwise hydrogenation (Figure 2c). In the competing pathway toward BHH, the ^*^C–^*^C coupling is exergonic (∆G < 0) at both 1^*^H and 8^*^H (Figure 2d), indicating a high tendency for side‐product formation under insufficient or excessive ^*^H conditions. Notably, at 4^*^H coverage, the coupling becomes thermodynamically unfavorable (∆G = +1.56 eV), effectively suppressing dimerization.

Collectively, these results underscore the pivotal role of surface ^*^H coverage in determining the reaction pathway over Cu_2_O. A moderate ^*^H level simultaneously lowers the kinetic barriers for DHMF formation and suppresses the thermodynamic driving force for dimerization (Figure 2e), thereby offering a dual‐function regulatory effect. These findings establish ^*^H coverage modulation as a key design principle for advancing efficient electrocatalysts in HMF electro‐hydrogenation.

Building upon these theoretical insights, developing a catalyst capable of achieving the optimal surface ^*^H coverage on Cu_2_O is therefore critical for the ECH of HMF for DHMF production. However, directly regulating the ^*^H distribution on the Cu_2_O is inherently challenging, as it is difficult to isolate surface effects while maintaining Cu(I) stability under reductive conditions. To address this challenge, we adopt a heterojunction strategy to modulate the interfacial electronic microenvironment of Cu_2_O by introducing a secondary component. The Co_3_O_4_ is selected as such a secondary component based on the following two considerations: its limited activity for HMF electroreduction under neutral conditions makes it catalytically silent yet electronically tunable,^[^ ^26^ ^]^ and its redox‐active nature allows it to undergo preferential reduction during operation,^[^ ^25^ ^]^ thereby stabilizing Cu(I) species and enhancing H_2_O dissociation.

**Figure 3:**
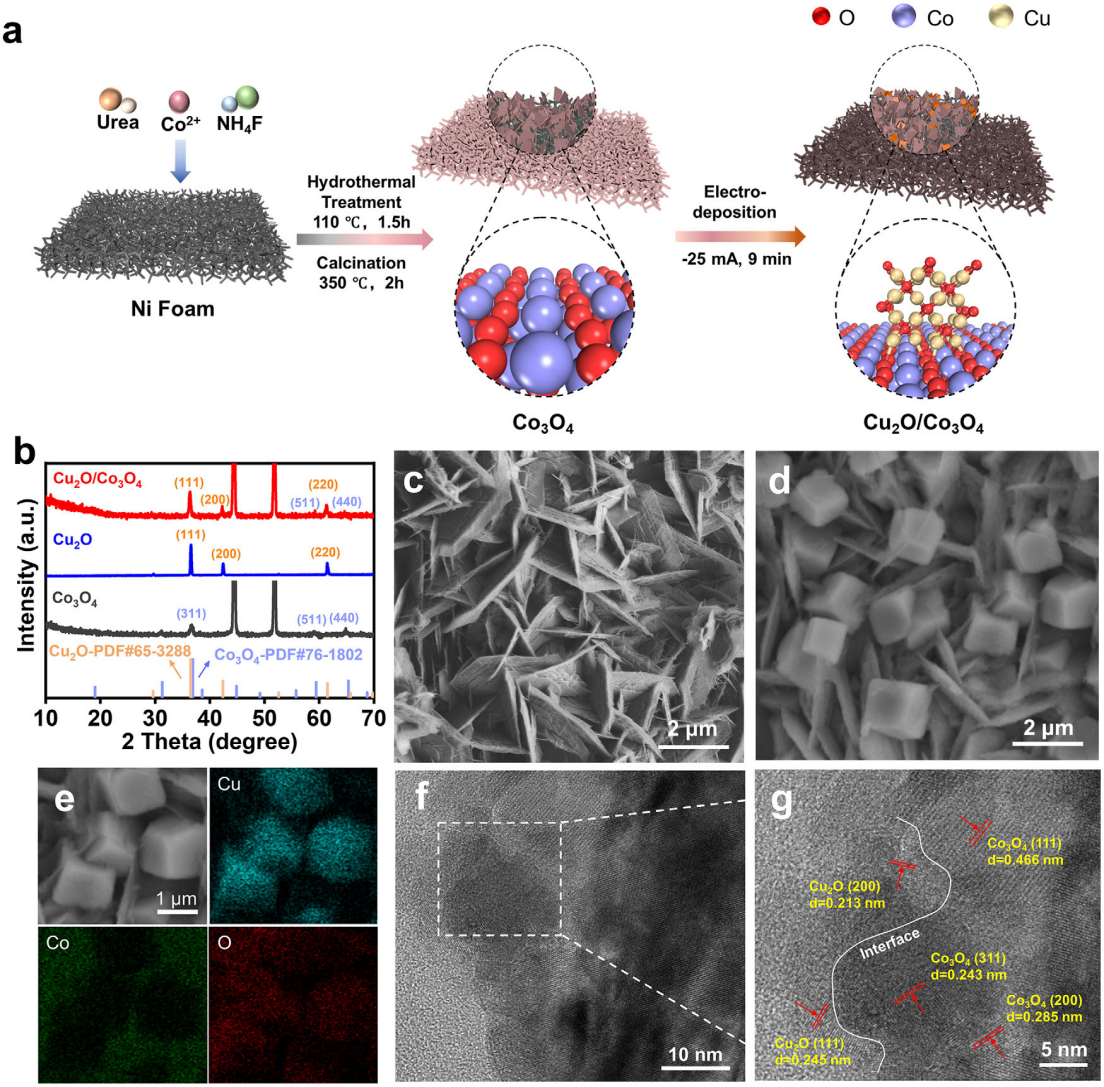
a) Schematic illustration of the synthetic route for Cu_2_O/Co_3_O_4_. b) XRD patterns of Co_3_O_4_, Cu_2_O, Cu_2_O/Co_3_O_4_. SEM image of c) Co_3_O_4_ and d) Cu_2_O/Co_3_O_4_. e) EDS mapping of Cu_2_O/Co_3_O_4_. f,g) HRTEM images of Cu_2_O/Co_3_O_4_.

Guided by this rationale, we synthesized a Cu_2_O/Co_3_O_4_ heterojunction catalyst via a hydrothermal–calcination–electrodeposition method (**Figure** **3**a) and systematically evaluated its structural and catalytic properties. X‐ray diffraction (XRD) patterns of the as‐synthesized Cu_2_O/Co_3_O_4_ (Figure 3b) display several characteristic diffraction peaks corresponding to the Cu_2_O (PDF #65‐3288) and Co_3_O_4_ (PDF #76‐1802), respectively, confirming the successful construction of the Cu_2_O/Co_3_O_4_ composited catalyst. The Raman spectra of the three catalysts were recorded as well (Figure S5, Supporting Information). The Raman shifts of 184, 458, 503, 595, 654 cm^−1^ in the Co_3_O_4_ spectrum are assigned to the F^1^_2g_, E_g_, F^2^_2g_, F^3^_2g_, A_1g_ modes of the crystalline Co_3_O_4_,^[^ ^27^, ^28^ ^]^ while the Raman shifts at 144, 213, 405, 630 cm^−1^ are attributed to the vibration modes of Cu_2_O.^[^ ^25^ ^]^ When these two components are combined to form the composited Cu_2_O/Co_3_O_4_, all the peaks of Co_3_O_4_ in the Cu_2_O/Co_3_O_4_ spectrum display blue shifts, implying that the Co_3_O_4_ might experience electron transfer due to the induction of Cu_2_O.^[^ ^29^, ^30^ ^]^

The scanning electron microscopy (SEM) image indicates that the Co_3_O_4_ exhibited a typical nanosheet array structure with an average thickness of 0.11 µm and a diameter of 3 µm (Figure 3c), while Cu_2_O exhibits a cubic particle structure with a size of 1 µm (Figure S6, Supporting Information). The SEM image of the Cu_2_O/Co_3_O_4_ displays abundant cubic particles uniformly dispersed on the nanosheet support (Figure 3d). Energy dispersive X‐ray spectroscopy (EDS) mapping further demonstrates that cobalt and copper are well distributed on sheets and cubes, respectively, confirming that the nanocube is Cu_2_O and the nanosheet is Co_3_O_4_ (Figure 3e). The high‐resolution transmission electron microscopy (HRTEM) images (Figure 3f,g) directly confirm an intimate Cu_2_O/Co_3_O_4_ heterointerface. The measured lattice spacings of 0.213 and 0.245 nm match the (200) and (111) planes of Cu_2_O, while 0.243, 0.285, and 0.466 nm index to the (311), (200), and (111) planes of Co_3_O_4_, respectively. Similar interfaces were observed across multiple fields of view; additional images and the corresponding FFT/SAED patterns supporting these assignments are provided in the (Figures S7 and S8, Supporting Information).

**Figure 4:**
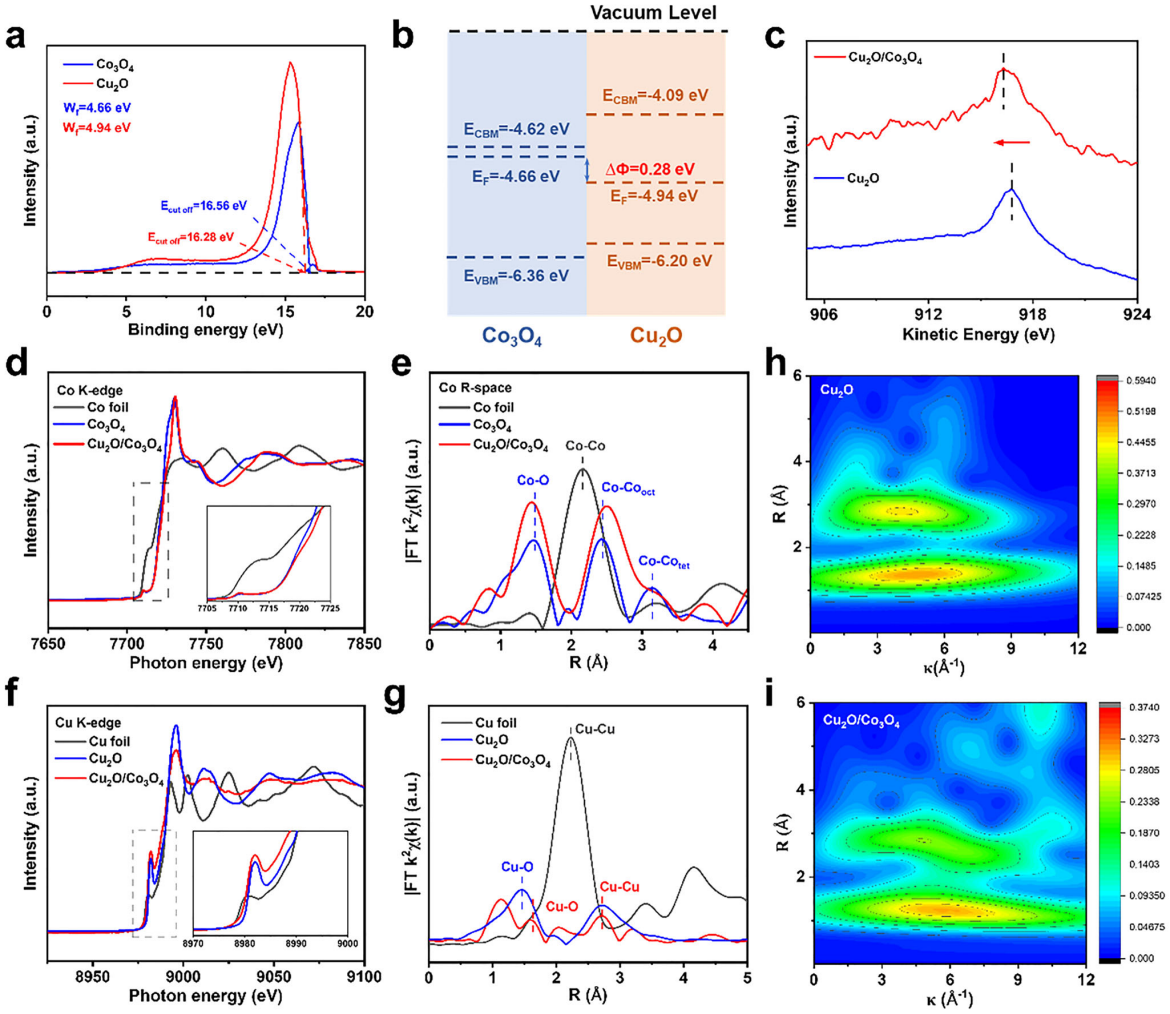
a) UPS spectra of Co_3_O_4_ and Cu_2_O. b) Schematic diagram of electron transfer and the corresponding energy band diagram of Co_3_O_4_ and Cu_2_O. c) Cu LMM spectrum of Cu_2_O/Co_3_O_4_ and Cu_2_O. d) Co K‐edge and (f) Cu K‐edge XANES spectra of the Cu_2_O/Co_3_O_4_. Corresponding FT transforms of k^2^‐weighted (e) Co K‐edge and (g) Cu K‐edge EXAFS spectra. h, i) Wavelet transform for the k^2^‐weighted EXAFS Cu edge.

Ultraviolet photoelectron spectroscopy (UPS) and UV–vis spectroscopy were subsequently applied to characterize the Cu_2_O/Co_3_O_4_ heterojunction. As shown in **Figures** **4**a and S9 (Supporting Information), the Cu_2_O exhibits a lower work function (−4.94 eV) than Co_3_O_4_ (−4.66 eV), suggesting a spontaneous electron transfer from Co_3_O_4_ to Cu_2_O upon contact. This interfacial charge redistribution implies the establishment of electronic interaction across the heterojunction,^[^ ^31^, ^32^ ^]^ and a simplified band alignment derived from the UPS and UV–vis results is illustrated in Figure 4b.

To probe the interfacial electronic interaction in the Cu_2_O /Co_3_O_4_ heterojunction, we first examined the valence states and electron redistribution using X‐ray photoelectron spectroscopy (XPS) and Auger electron spectroscopy (AES). As shown in Figure S10 (Supporting Information), the Co 2p_3/2_ peak shifts positively and the Cu 2p_3/2_ peak shifts negatively upon heterojunction formation, indicating electron transfer from Co to Cu.^[^ ^33^ ^]^ This charge redistribution is further supported by Cu LMM Auger analysis (Figure 4c), where the Cu(I) species exhibit a shift to lower kinetic energy, suggesting an increased electron density around Cu sites.^[^ ^34^ ^]^

To gain deeper insights into the coordination environment and electronic structure, X‐ray absorption near‐edge structure (XANES) and extended X‐ray absorption fine structure (EXAFS) measurements were conducted. The Cu K‐edge XANES of Cu_2_O/Co_3_O_4_ lies between those of Cu foil and pristine Cu_2_O (Figure 4f), indicating a mixed Cu(0)/Cu(I) state.^[^ ^35^ ^]^ Concurrently, the Co K‐edge in the heterojunction shifts positively relative to the pristine Co_3_O_4_ (Figure 4d), corroborating the electron donation from Co to Cu.^[^ ^36^ ^]^ These trends align well with XPS and AES findings. EXAFS analysis further reveals interfacial structural distortions induced by charge transfer. The Co─O bond length in Cu_2_O/Co_3_O_4_ contracts from 1.48 to 1.44 Å, while the Cu─O bond length expands from 1.46 to 1.59 Å (Figure 4e,g; Figure S11, Supporting Information), reflecting electron depletion and enrichment around Co and Cu centers, respectively.^[^ ^37^ ^]^ These changes are consistent with electronic modulation at the heterojunction. Moreover, wavelet transform (WT) analysis at the Cu K‐edge (Figure 4h,i) exhibited slightly shift Cu‐Cu and Cu‐O features (5.2 and 6.5 Å^−1^) compared to the pristine Cu_2_O (4.2 and 4.7 Å^−1^), further confirming the altered coordination environments modulated by Co_3_O_4_.^[^ ^37^, ^38^ ^]^ The absence of Cu‐O‐Co features in WT spectra suggests that the interfacial interaction arises from electronic coupling rather than direct chemical bonding.^[^ ^39^ ^]^ Collectively, these results suggest that Co_3_O_4_ could modulate the electronic structure of Cu_2_O, which may help improve its catalytic performance.

**Figure 5:**
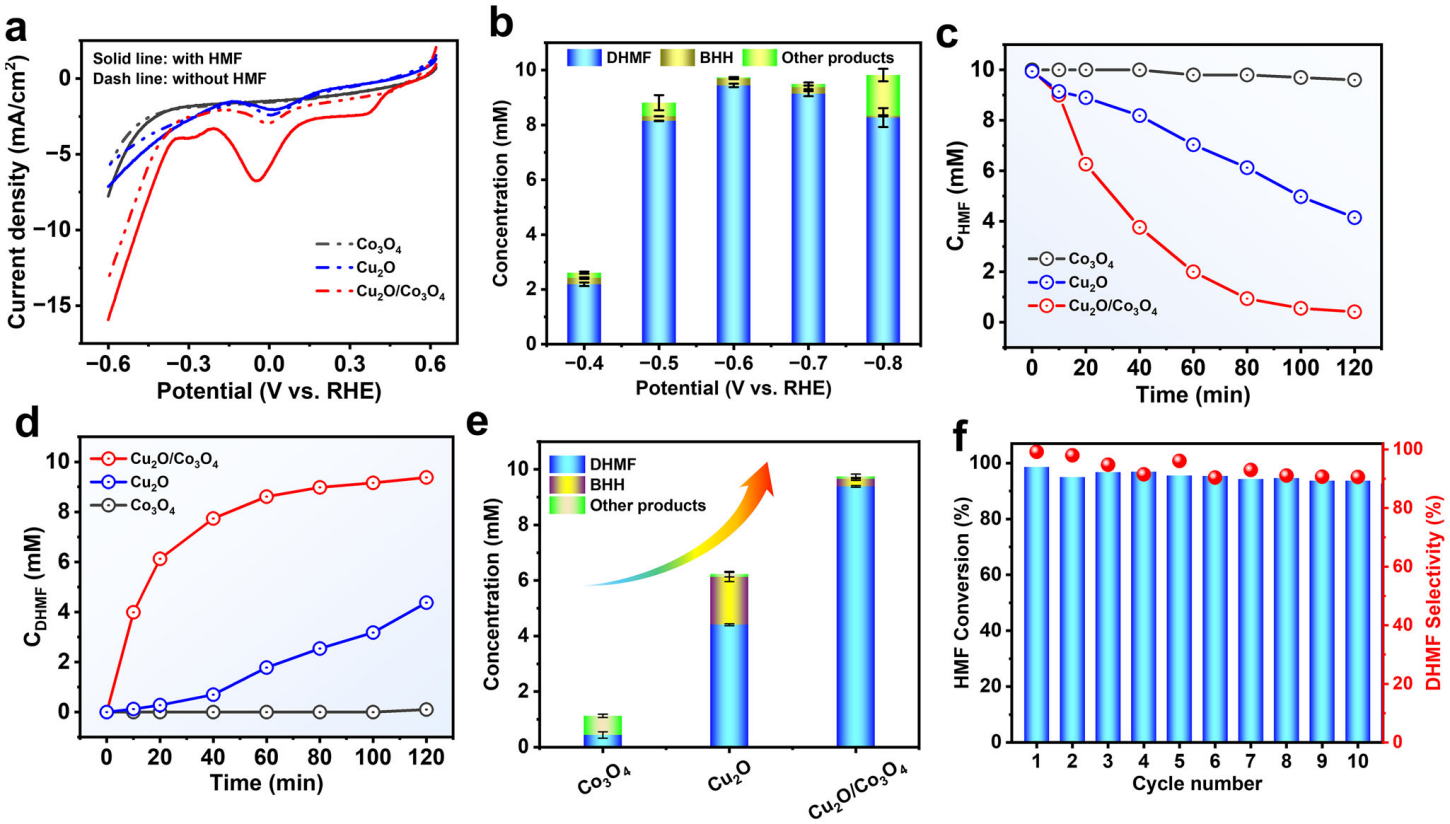
a) LSV curves of Cu_2_O/Co_3_O_4_, Cu_2_O, and Co_3_O_4_ in 0.1 m N_2_ saturated Na_2_SO_4_ with and without 10 mm HMF with a scan rate of 10 mV s^−1^. b) The comparison of product distribution over Cu_2_O/Co_3_O_4_ under the potential window from −0.4 to −0.8 V versus RHE c,d) Time‐dependent concentration changes of HMF and DHMF. e) The comparison of product distribution over Cu_2_O/Co_3_O_4_, Cu_2_O, and Co_3_O_4_ at −0.6 V versus RHE. f) Cycle‐dependent HMF conversion and DHMF selectivity over Cu_2_O/Co_3_O_4_ at −0.6 V versus RHE.

To assess the intrinsic electrocatalytic activity of the as‐synthesized Cu_2_O/Co_3_O_4_ catalyst, linear sweep voltammetry (LSV) measurements were conducted under neutral conditions. As shown in **Figure** **5**a, the Cu_2_O/Co_3_O_4_ exhibits a significantly higher current density than the pristine Cu_2_O and Co_3_O_4_ during the electrochemical process without adding HMF, indicating that the introduction of Co_3_O_4_ effectively enhanced the water dissociation kinetics of Cu_2_O. Upon adding HMF, the Cu_2_O/Co_3_O_4_ electrode shows the most pronounced enhancement, suggesting that the ECH of HMF is thermodynamics more favorable than the hydrogen evolution reaction. Consistent with this, Tafel analyses (Figure S12, Supporting Information) show smaller slopes for Cu_2_O/Co_3_O_4_ than for Cu_2_O for both hydrogen evolution reaction (HER) and HMF reduction reaction (HMFRR), reflecting faster charge‐transfer kinetics on Cu_2_O/Co_3_O_4_.

To identify the optimal operational condition, chronoamperometry measurements were conducted, and the corresponding product distributions were analyzed (Figures S13–S15, Supporting Information). As shown in Figure 5b, Cu_2_O/Co_3_O_4_ maintained >80% HMF conversion and DHMF selectivity between −0.50 and −0.80 V versus RHE, with peak performance at −0.60 V versus RHE. A lower potential (−0.4 and −0.5 V vs RHE) may lead to a lower HMF conversion and higher BHH selectivity, while a higher potential (−0.8 V vs RHE) decreased the selectivity to DHMF due to the further hydrodeoxygenation of DHMF or hydrogenation of the furan ring in HMF or DHMF (denoted as other products in Figure 5b).

We next benchmarked all catalysts at a representative potential of −0.60 V versus RHE. As shown in Figure 5c,d, the Cu_2_O/Co_3_O_4_ heterojunction catalyst achieves 97% HMF conversion and 97% 2,5‐dihydroxymethylfuran (DHMF) selectivity, markedly outperforming the pristine Cu_2_O (62% conversion, 71% selectivity). In contrast, the Co_3_O_4_ alone exhibits negligible catalytic activity (11.3% HMF conversion), even lower than that of bare nickel foam (20.1%) (Figure S17, Supporting Information), reinforcing its role as a catalytically inert but electronically tunable cocatalyst. Moreover, the formation of the dimeric byproduct 5,5′‐bis(hydroxymethyl)hydrofuroin (BHH) typically associated with ketyl radical coupling is significantly suppressed in the Cu_2_O/Co_3_O_4_ system, with selectivity reduced from 28% (Cu_2_O) to below 3% (Figure 5e; Figure S16, Supporting Information), suggesting that the Co_3_O_4_ modulates the Cu_2_O surface to steer the reaction pathway toward DHMF formation. The superiority of the heterojunction can be further supported by a kinetic analysis. As shown in Figure S18 (Supporting Information), the Cu_2_O/Co_3_O_4_ catalyst displays a reaction rate constant (*k*) of 2.87 × 10^−2^ min^−1^ markedly higher than those of Cu_2_O (7.09 × 10^−3^ min^−1^) and Co_3_O_4_ (3.56 × 10^−4^ min^−1^).

Targeted controls further corroborate the interfacial origin of this enhancement. A physical mixture of Cu_2_O + Co_3_O_4_ showed poor performance (9.9% conversion, 27.9% selectivity; Figure R10b), while Cu_2_O on inert oxides that minimized charge transfer (SiO_2_ and ZrO_2_) retained relatively high DHMF selectivity (88.5% and 95.8%) but exhibited much lower conversion (31.4% and 33.1%) (Figure S19, Supporting Information). Collectively, these results rule out simple compositional/proximity effects and identify electronic modulation at the Cu_2_O/Co_3_O_4_ interface as the key factor governing activity and byproduct suppression.

The stability of Cu_2_O/Co_3_O_4_ was verified via 10‐cycle recyclability tests (20 h total). As shown in Figure 5f, the catalyst retained >90% of its original conversion and selectivity, with minimal current degradation (Figure S20, Supporting Information). Post‐electrolysis inductively coupled plasma (ICP) analysis of the electrolyte indicated low leaching (Cu 0.427 mg L^−1^, 0.15%; Co 0.129 mg L^−1^, 0.046%) (Table S1, Supporting Information), confirming stable composition under operating conditions. SEM images (Figure S21, Supporting Information) reveal that the catalyst maintained its structure and morphology after 10 cycles, indicating high structural stability.

Finally, relative to recent non‐noble benchmarks, Cu_2_O/Co_3_O_4_ was competitive by delivering up to 97%/97% conversion/selectivity in a simple neutral Na_2_SO_4_ electrolyte—without PBS/borate buffers or soluble mediators—via a heterojunction strategy that electronically tuned ^*^H supply (Table S2, Supporting Information). This positioned the interface‐engineered Cu‐based system as a practical, scalable platform for selective HMF electro‐hydrogenation.

To directly track the Cu valence under working conditions, we performed quasi in situ XPS on the Cu LMM region for pristine Cu_2_O and the Cu_2_O/Co_3_O_4_ heterojunction at open‐circuit and a series of cathodic potentials. As shown in Figure S22 (Supporting Information), a Cu° component (≈918.6 eV) appears already at −0.30 V and grows at −0.60/−0.70 V versus RHE on pristine Cu_2_O, indicating progressive reduction of Cu^+^. In contrast, Cu_2_O/Co_3_O_4_ remains Cu^+^‐dominated with only weak Cu^0^ even at −0.80 V versus RHE. This potential‐resolved comparison demonstrates that the Co_3_O_4_ interface suppressed over‐reduction of Cu⁺ via a redox‐buffering effect. Complementary time‐resolved XPS during HMF electro‐hydrogenation showed a persistent Cu^+^ signature in Cu 2p (Figure S23, Supporting Information), while the Co 2p envelope shifted toward higher Co^2+^ content (from 41.5% at 0 min to a dominant fraction at the end; Figure S24 and Table S3, Supporting Information), consistent with preferential reduction of Co_3_O_4_ under bias. This behavior was further corroborated by H_2_‐temperature programmed desorption (TPD) analysis (Figure S25, Supporting Information), which confirmed the enhanced reducibility of Co_3_O_4_ and its ability to stabilize Cu^+^ species in the heterojunction.

**Figure 6:**
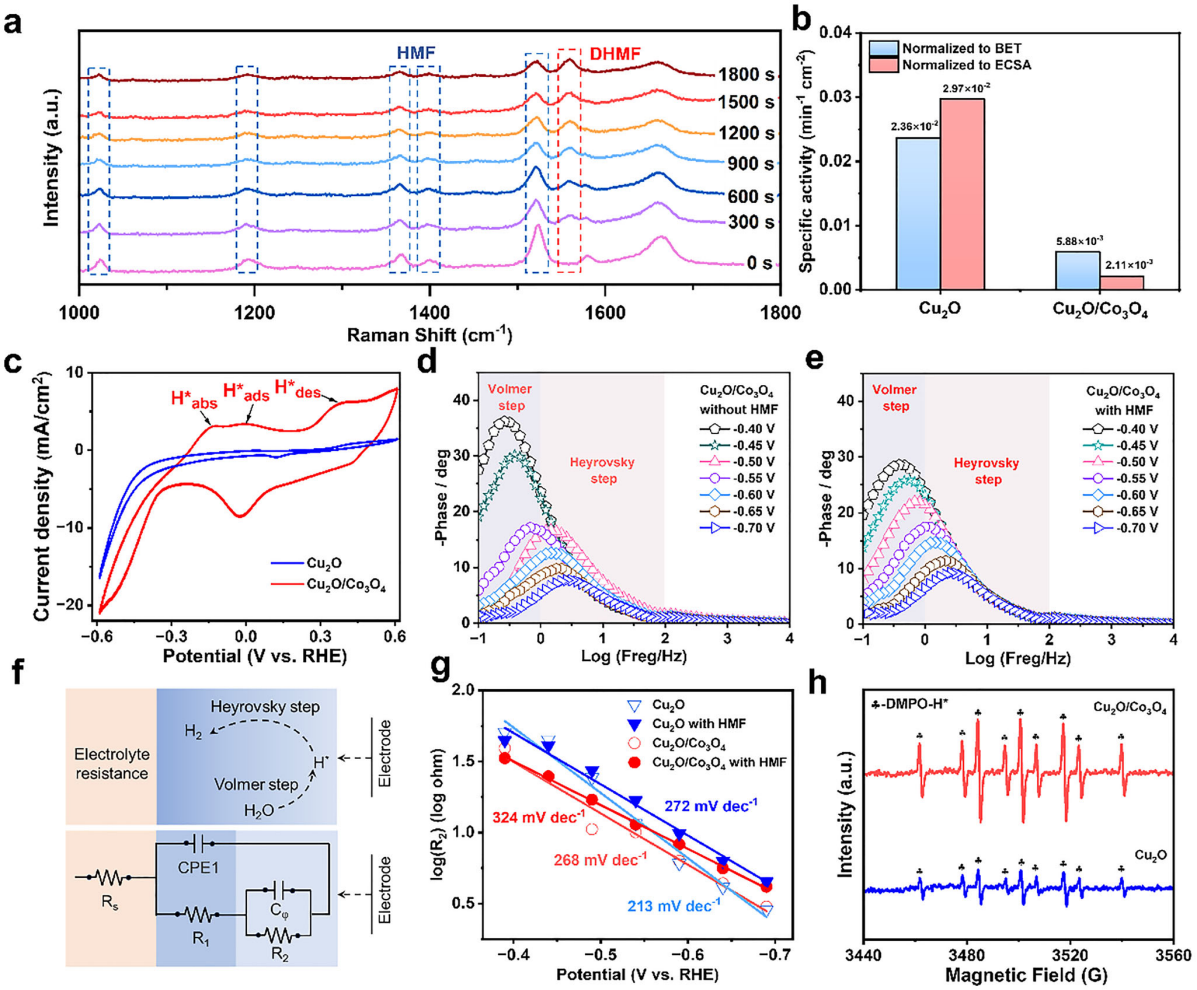
a) The change of in situ Raman spectroscopy of Cu_2_O/Co_3_O_4_ at −0.6 V versus RHE in the 0.1 m Na_2_SO_4_ electrolyte with 50 mm HMF. b) Normalized rate constants for Cu_2_O/Co_3_O_4_ and Cu_2_O, relative to their respective BET and ECSA. c) CV curves of Cu_2_O/Co_3_O_4_, Cu_2_O in 0.1 m Na_2_SO_4_ with a scan rate of 10 mV s^−1^. Bode plots for Cu_2_O/Co_3_O_4_ in d) 0.1 m Na_2_SO_4_ and e) 0.1 m Na_2_SO_4_ with 50 mm HMF. f) The equivalent circuit model used in the fitting of the electrochemical impedance spectra and schematic diagram of the proposed HER. g) EIS‐derived plots of the Cu_2_O/Co_3_O_4_ and Cu_2_O catalysts obtained from the ^*^H adsorption resistance R_2_. h) Quasi‐in situ EPR trapping for hydrogen radical over Cu_2_O/Co_3_O_4_ and Cu_2_O.

To gain mechanistic insight into the electrochemical hydrogenation of HMF, in situ Raman spectroscopy was conducted in 0.1 m Na_2_SO_4_ containing 10 mm HMF. As shown in **Figure** **6**a, during potentiostatic electrolysis at −0.6 V versus RHE, the characteristic Raman bands of HMF at 1026, 1197, 1369, 1403, and 1523 cm^−1^ progressively diminish (Figure S26, Supporting Information), indicating continuous substrate consumption. Concurrently, a new band at 1560 cm^−1^, attributed to DHMF, emerges and intensifies over time, providing direct spectroscopic evidence of product formation on the Cu_2_O/Co_3_O_4_ surface. A similar spectral evolution is observed under varying applied potentials (Figure S27, Supporting Information), further confirming the potential‐dependent conversion of HMF to DHMF at the heterointerface.

To uncover the origins of the enhanced catalytic activity of Cu_2_O/Co_3_O_4_, the electrochemical active surface area (ECSA) was evaluated by measuring the double‐layer capacitance (C_dl_) via cyclic voltammetry (Figure S28a–c, Supporting Information). The Cu_2_O/Co_3_O_4_ heterojunction exhibits a C_dl_ of 13.6 mF cm^−2^, markedly higher than that of Co_3_O_4_ (12.9 mF cm^−2^) and Cu_2_O (0.239 mF cm^−2^), indicating an expanded active electrochemical area for HMFRR (Figure S28d, Supporting Information).^[^ ^40^ ^]^ Notably, BET surface area analysis shows a similar trend, suggesting that the ECSA increase is partly attributable to enhanced textural properties of the heterojunction (Figure S29, Supporting Information). To further assess the intrinsic catalytic activity, the reaction rate constants k) were normalized by both ECSA and BET surface area. Figure 6b and Figure S30 (Supporting Information) reveal that Cu_2_O exhibited the highest specific activity, confirming its role as a primary active site, while Co_3_O_4_ acts as a cocatalyst to enhance the catalytic activity of Cu_2_O toward ECH of HMF.

We then systematically examined the behavior of ^*^H on the catalyst, given its pivotal role in determining the selectivity for the ECH of HMF. As shown in the cyclic voltammetry (CV) profiles recorded in 0.1 m Na_2_SO_4_ (Figure 6c), the Cu_2_O/Co_3_O_4_ exhibits three anodic features centered at −0.13, 0, and 0.4 V versus RHE, corresponding to ^*^H absorption (^*^H_abs_), adsorption (^*^H_ads_), and desorption (^*^H_des_), respectively.^[^ ^41^, ^42^, ^43^, ^44^, ^45^, ^46^ ^]^ In contrast, Cu_2_O exhibits no discernible ^*^H‐related peaks, suggesting its limited H_2_O dissociation ability. This observation highlights the critical role of Co_3_O_4_ in promoting H_2_O dissociation and enriching ^*^H coverage on the surface of Cu_2_O. Furthermore, upon the introduction of HMF, the ^*^H adsorption and desorption peaks in the Cu_2_O/Co_3_O_4_ CV profile weaken significantly (Figure S31, Supporting Information), likely due to the ^*^H consumption during the ECH of HMF.^[^ ^8^ ^]^ This observation suggests that Cu_2_O/Co_3_O_4_ efficiently catalyzes the ECH of HMF via an ^*^H addition mechanism.

To probe water activation, H/D substitution experiments were conducted. Replacing H_2_O with D_2_O led to a cathodic shift of the LSV curves; notably, the magnitude of this shift was much smaller for Cu_2_O/Co_3_O_4_ compared to pristine Cu_2_O (Figure S32, Supporting Information). This observation indicates that the heterojunction suffered from a weaker isotope penalty and thus facilitated water dissociation more efficiently at the relevant potentials. As shown in Figure S33 (Supporting Information), the corresponding kinetic isotope effect (KIE) values were 1.4 for Cu_2_O/Co_3_O_4_ and 2.4 for Cu_2_O. The substantially smaller KIE on the heterojunction demonstrates its ability to lower the barrier for water activation and to sustain higher ^*^H coverage than Cu_2_O.

In situ electrochemical impedance spectroscopy (EIS) analysis was conducted over a potential range of −0.8–−0.4 V versus RHE to investigate the role of surface ^*^H species in the ECH of HMF. As shown in the Bode plots (Figure 6d), Cu_2_O/Co_3_O_4_ exhibits a dominant peak in the low‐frequency region (<1 Hz) under low overpotentials, which is attributed to the Volmer step (H_2_O + e^1^ → H + OH^−^), the key step responsible for surface ^*^H generation.^[^ ^8^, ^47^ ^]^ This behavior suggests that the sluggish H_2_O dissociation limits the rate of surface ^*^H supply at lower potentials. With increasing potential, the peak position progressively shifts toward the mid‐frequency region (1–100 Hz), corresponding to the Heyrovsky step (^*^H + H_2_O + e^−^ → H_2_ + OH^−^), which means that the hydrogen evolution becomes the main reaction.^[^ ^47^ ^]^ Upon adding HMF, the Bode peaks for Cu_2_O/Co_3_O_4_ shift toward higher frequencies and display markedly reduced intensity (Figure 6e), indicating a lower interfacial resistance and accelerated kinetics. This is likely due to the rapid consumption of ^*^H by the aldehyde group of HMF, which breaks the Volmer equilibrium and redirects the reaction pathway toward HMF hydrogenation.^[^ ^48^ ^]^ By contrast, Cu_2_O alone exhibits a Bode peak consistently located in the mid‐frequency region. Notably, after HMF addition, though the overall impedance decreased, the peak shifts toward lower frequencies, suggesting a reduction in reaction kinetics (Figure S34, Supporting Information). This behavior likely stems from competitive adsorption between HMF and H_2_O, thereby suppressing ^*^H formation and impairing the hydrogenation process of HMF. In conclusion, these findings collectively support a ^*^H‐mediated hydrogen atom transfer (HAT) mechanism and underscore the essential role of Cu_2_O/Co_3_O_4_ in facilitating ^*^H accumulation and utilization.

A double‐parallel equivalent circuit was employed to simulate the EIS results (Figure 6d,e; Figures S34 and S35, Supporting Information), with fitting parameters provided in Tables S4 and S5 (Supporting Information). As depicted in the equivalent circuit (Figure 6f), the cathodic resistance is divided into three components: (I) charge transfer resistance (R_1_), (II) intermediates adsorption resistance (R_2_), and (III) electrolyte resistance (R_s_).^[^ ^8^, ^48^ ^]^ Notably, R_2_ serves as an indicator of localized ^*^H enrichment, as higher ^*^H coverage inhibits further adsorption, leading to increased R_2_ values.^[^ ^49^, ^50^ ^]^ Accordingly, the EIS‐derived slope, calculated by plotting Log (R_2_) versus potential, provides a quantitative measure of localized ^*^H enrichment.^[^ ^51^ ^]^ As shown in Figure 6g, the Cu_2_O/Co_3_O_4_ exhibits a slope of 268 mV dec^−1^, significantly higher than that of single Cu_2_O (213 mV dec^−1^), indicating superior ^*^H enrichment at the heterojunction.^[^ ^42^, ^44^, ^49^, ^52^ ^]^ Upon the addition of HMF, the slope increases for both catalysts (from 213 to 272 mV dec^−1^ for Cu_2_O and from 268 to 324 mV dec^−1^ for Cu_2_O/Co_3_O_4_), likely due to the ^*^H consumption during HMF reduction, which further promotes ^*^H production and enrichment.

To further probe the generation and role of surface‐bound H species, quasi‐in situ electron paramagnetic resonance (EPR) spectroscopy was performed using 5,5‐dimethyl‐1‐pyrroline‐N‐oxide (DMPO) as a spin‐trapping agent. As shown in Figure 6h, both Cu_2_O/Co_3_O_4_ and single Cu_2_O catalysts display ^*^H signals, with significantly stronger signals for the Cu_2_O/Co_3_O_4_. Upon introduction of 50 mm HMF, the ^*^H signal disappears for Cu_2_O but remains clearly visible for Cu_2_O/Co_3_O_4_ (Figure S36, Supporting Information). These results indicate that Co_3_O_4_ facilitates H_2_O dissociation and enhances ^*^H coverage on the Cu_2_O surface, thereby sustaining ^*^H availability during HMF reduction. CV measurements (Figure S37, Supporting Information) confirm that tert‐butyl alcohol (t‐BuOH) acts as an H scavenger.^[^ ^53^ ^]^ Upon its introduction into the reaction system, HMF conversion, DHMF yield, and the reaction rate decrease significantly (Figure S38, Supporting Information), highlighting the essential role of ^*^H in the electrocatalytic hydrogenation of HMF.

**Figure 7:**
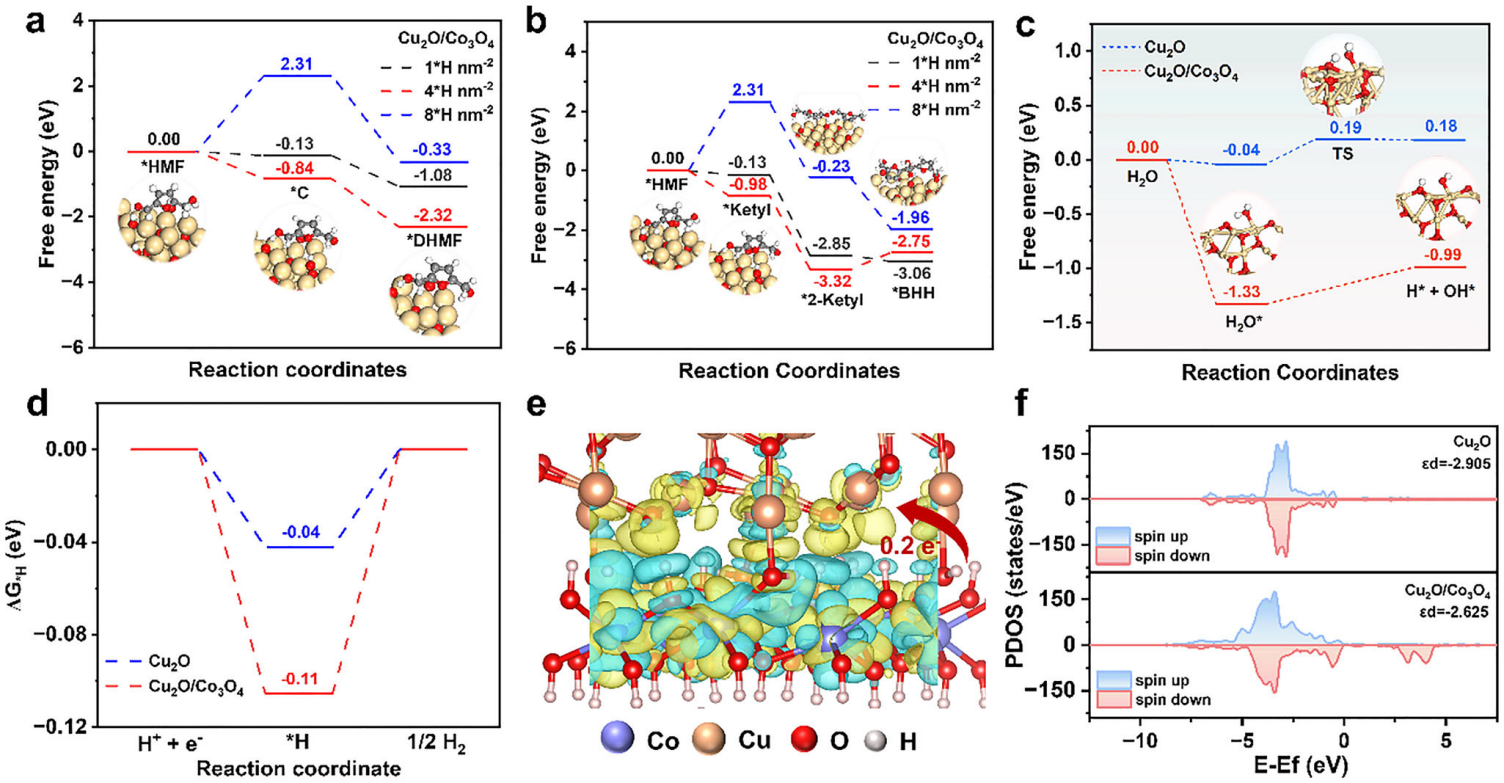
Calculated free energy profiles for HMF hydrogenation to a) DHMF and b) BHH on Cu_2_O/Co_3_O_4_ using the HAT reaction mechanism under different hydrogen coverage. c) Calculated free energy of water dissociation on Cu_2_O and Cu_2_O/Co_3_O_4_. d) Hydrogen adsorption Gibbs free energy diagram of Cu_2_O and Cu_2_O/Co_3_O_4_. e) Charge density difference at the interface of Cu_2_O/Co_3_O_4_, the blue and yellow regions reflect electron‐depletion state and electron‐accumulation area, respectively. f) PDOS of d orbitals of Cu_2_O and Cu_2_O/Co_3_O_4_ heterostructure.

To gain atomic‐scale insights into how the Cu_2_O/Co_3_O_4_ modulated the localized ^*^H enrichment and enhanced 2,5‐dihydroxymethylfuran (DHMF) selectivity, DFT calculations were conducted. After identifying a flat‐lying adsorption configuration of HMF on the heterojunction as the most stable geometry (Figures S39–S41, Supporting Information), we evaluate the energy profiles for two representative hydrogenation routes under varying ^*^H coverages. At a moderate ^*^H coverage of 4 ^*^H nm^−2^, the ECH of HMF to DHMF proceeds spontaneously, exhibiting a negative Gibbs free energy change (**Figure** **7**a), whereas the competing dimerization route toward 5,5′‐bis(hydroxymethyl)hydrofuroin (BHH) remains thermodynamically unfavorable (Figure 7b). These results are consistent with experimental observations—Cu_2_O/Co_3_O_4_ delivers >97% DHMF selectivity and suppresses BHH formation to <3%—and highlight the critical role of surface ^*^H coverage in dictating product distribution at the heterojunction (Figure S42, Supporting Information).

Figure 7c presents the free energy diagram for water dissociation on Cu_2_O and Cu_2_O/Co_3_O_4_ catalysts. The heterojunction enables spontaneous H_2_O dissociation with no energy barrier, whereas Cu_2_O requires an activation energy of 0.23 eV, indicating a more favorable kinetics for ^*^H generation on Cu_2_O/Co_3_O_4_ and ensuring a continuous hydrogen supply during catalysis. To further investigate the bond activation process, crystal orbital Hamilton population (COHP) analysis was conducted. The integrated COHP (ICOHP) value for the O─H bond decreases from −3.42 on Cu_2_O to −3.39 on the heterojunction (Figure S43, Supporting Information), indicating a weakened bond that facilitates water cleavage and enhances ^*^H coverage. Additionally, HER free energy diagrams (Figure 7d) reveal that the heterojunction exhibits a stronger ^*^H binding (−0.11 eV) than Cu_2_O (‐0.04 eV), suggesting more efficient hydrogen stabilization. This optimized adsorption effectively suppresses competing H_2_ evolution and promotes selective hydrogen transfer to HMF.

To uncover the electronic basis for this improvement, we performed charge density difference analysis. As shown in Figure 7e, substantial interfacial charge redistribution occurs at the interface, with net electron transfer (0.20 e^−^) from Co_3_O_4_ to Cu_2_O according to Bader analysis, suggesting that Co_3_O_4_ effectively modulates the local electronic structure of Cu sites. This modulation is further supported by partial density of states (PDOS) analysis (Figure 7f), which reveals a notable upward shift in the Cu d‐band center from −2.905 eV (Cu_2_O) to −2.625 eV in the heterojunction. This shift facilitates stronger H_2_O adsorption and promotes H─O bond activation, thereby promoting ^*^H generation and enhancing the hydrogenation of HMF.

In summary, these theoretical insights support a mechanistic scenario wherein the incorporation of Co_3_O_4_ facilitates water dissociation and regulates ^*^H coverage on Cu_2_O, enabling precise pathway control in HMF hydrogenation. This interfacial synergy not only stabilizes Cu⁺ species but also tunes the electronic structure of Cu sites, collectively facilitating high DHMF selectivity via a HAT mechanism.

## 3 Conclusion

In conclusion, this work identifies surface hydrogen (^*^H) coverage as a key factor governing product selectivity in the ECH of HMF. DFT calculations reveal that moderate ^*^H coverage on the Cu_2_O selectively favors DHMF formation over dimeric byproducts. Guided by theoretical calculations, the Cu_2_O/Co_3_O_4_ heterojunction catalyst is developed and evaluated in the ECH of HMF, achieving 97% HMF conversion and 97% DHMF selectivity under neutral conditions—significantly outperforming the pristine Cu_2_O. Mechanistic studies combined with theoretical simulations demonstrate that Co_3_O_4_ serves as a redox‐active cocatalyst to stabilize Cu(I) species and modulate the electronic structure of Cu_2_O, thereby promoting H_2_O dissociation kinetics and enabling effective tuning of surface ^*^H coverage to achieve high DHMF selectivity. This study highlights a rational heterojunction engineering approach to simultaneously stabilize active centers and regulate hydrogen dynamics, providing generalizable insights for the design of highly selective biomass electro‐reduction catalysts under mild conditions.

## Conflict of Interest

The authors declare no conflict of interest.

## Supporting information



Supporting Information

## Data Availability

The data that support the findings of this study are available from the corresponding author upon reasonable request.
