# Supplementary material for: Optimizing Surface *H Coverage over Cu2O/Co3O4 Heterojunction Enables Efficient Neutral Electrocatalytic Hydrogenation of 5‐hydroxymethylfurfural to 2,5‐dihydroxymethylfuran
Source: Adv Sci (Weinh). 2025 Sep 26;12(47):e13460. doi: 10.1002/advs.202513460 (PMC12713019; doi:10.1002/advs.202513460)
Supplement: Supplementary file 1 — Supporting Information [file ADVS-12-e13460-s001.docx]

Supporting Information

**Optimizing surface *H coverage over Cu_2_O/Co_3_O_4_ heterojunction enables efficient neutral electrocatalytic hydrogenation of 5-hydroxymethylfurfural to 2,5-dihydroxymethylfuran**

Yun Ge, ^+[a]^ Wei Wang, ^+[a]^ Xiao-Qiang Pan, ^+[a]^ Jia-Wei Huang, ^[a]^ Jie-Jie Chen, ^[a]^ Wu-Jun Liu, *^[a]^ Yuqin Zou, *^[b]^ Han-Qing Yu*^[a]^

[a] Yun Ge, Wei Wang, Xiao-Qiang Pan, Jia-Wei Huang, Prof. Jie-Jie Chen, Prof. Wu-Jun Liu, Prof. Han-Qing Yu
State Key Laboratory of Advanced Environmental Technology, Department of Environmental Science and Engineering
University of Science & Technology of China
Hefei, 230026, China
E-mail: [liuwujun@mail.ustc.edu.cn](mailto:liuwujun@mail.ustc.edu.cn), [hqyu@ustc.edu.cn](mailto:hqyu@ustc.edu.cn)

[b] Prof. Yuqin Zou
State Key Laboratory of Chemo/Bio-Sensing and Chemometrics, College of Chemistry and Chemical Engineering, Advanced Catalytic Engineering Research Center of the Ministry of Education
Hunan University
Changsha, Hunan, 410082, P. R. China

E-mail: yuqin_zou@hnu.edu.cn

[+] These authors contributed equally.

Experimental Section

**Chemical and materials**

Cobalt nitrate hexahydrate (Co(NO_3_)_2_·6H_2_O), ammonium fluoride (NH_4_F), urea (CH_4_N_2_O), sodium sulfate (Na_2_SO_4_), and copper sulfate pentahydrate (CuSO_4_•5H_2_O) were purchased from Sinopharm Chemical Reagent Co., China. Ni foam (99.95% purity) was purchased from Saibo Electrochemistry Company. 5-hydroxymethylfurfural (HMF, 99%) and 2,5-dihydroxymethylfuran (DHMF, 99%) were purchased from Sigma-Aldrich.

**Catalyst synthesis**

**Synthesis of Co_3_O_4_**

Firstly, Co(OH)_2_ growing on a Ni foam substrate was synthesized via the hydrothermal method. In a typical procedure, Co(NO_3_)_2_·6H_2_O (0.436 g, 1.5 mmol), NH_4_F (0.111 g, 3 mmol), and urea (0.450 g, 7.5 mmol) were dissolved in 50 mL of water and stirred to form a clear solution. Nickel foam (1 cm×2.5 cm) was cleaned with ethanol and 1 M HCl to remove the surface organics and oxides. The aqueous solution and the Ni foam were transferred to a 100 mL Teflon-lined stainless-steel autoclave, which was sealed and maintained at 110 ℃ for 1.5 h. After cooling to room temperature, the pink film on the nickel foam was taken out and rinsed several times with pure water. Next, the sample was annealed in air at 623 K for 2 h in a muffle furnace to obtain Co_3_O_4_.

**Synthesis of Cu_2_O**

Cu_2_O was synthesized as follows. In a typical procedure, 170 mL of H_2_O was first mixed with 10 mL of 0.68 M CuSO_4_ and 3.0 g PVP (k-30, MW=30000) in a round-bottomed glass flask. The mixture was stirred for about 15-20 min, and then 10 mL of 0.74 M sodium citrate and 1.2 M anhydrous sodium carbonate solution was added to the above solution dropwise. After about 10 min, 10 mL of 1.4 M glucose solution was slowly dropped into it. The solution was kept in a water bath at a temperature of 80 °C for 2 h, and then cooled to room temperature naturally. The brick red precipitate was filtered off, washed several times with distilled water and absolute alcohol, and finally dried in a vacuum at 60 °C for 8 h. 5 mg of the as-prepared Cu_2_O was sonicated in 0.4 mL ethanol added with 10 μL Nafion (5 wt%) and then drop-casted onto Ni foam to form Cu_2_O.

**Synthesis of Cu_2_O/Co_3_O_4_**

Firstly, Co(OH)_2_ growing on the Ni foam substrate was synthesized via the hydrothermal method. In a typical procedure, Co(NO_3_)_2_·6H_2_O (0.436 g, 1.5 mmol), NH4F (0.111 g, 3 mmol), and urea (0.450 g, 7.5 mmol) were dissolved in 50 mL water and stirred to form a clear solution. Ni foam (1 cm×2.5 cm) was cleaned with ethanol and 1 M HCl to remove the surface organics and oxides. The aqueous solution and the Ni foam were transferred to a 100 mL Teflon-lined stainless-steel autoclave, which was sealed and maintained at 110 ℃ for 1.5 h. After cooling to room temperature, the pink film on the nickel foam was taken out and rinsed several times with pure water. Next, the sample was annealed in air at 623 K for 2 h in a muffle furnace to obtain Co_3_O_4_. Afterward, the Cu_2_O was loaded on the Co_3_O_4_ via an electro-deposition method described as follows: a typical deposition process proceeded under a galvanostatic state at -25 mA for 9 min in 80 mL solution containing 10 mM CuSO_4_ and 50 mM Na_2_SO_4_. Finally, the Cu_2_O/Co_3_O_4_ was obtained and used as an electrode without further treatment.

**Material characterizations**

The morphology and elemental mapping images of the electrodes were characterized using scanning electron microscopy (SEM, Hitachi SU8220, Japan) equipped with energy-dispersive X-ray spectroscopy (EDS, Aztec, Oxford Instrument plc, UK) detector. The lattice spacing was measured on high-resolution transmission electron microscopy (HRTEM, Talos F200X, Thermo Scientific Inc., USA). The phase and crystal structure were examined on powder X-ray diffraction (XRD, TTR-III, Rigaku Co., Japan) and Raman spectrometer (LABRAM HR EVO, Horiba Co., Japan). In-situ Raman was carried out in a homemade electrolyte cell. Ultraviolet photoelectron spectroscopy (UPS) and X-ray photoelectron spectroscopy (XPS) measurements were carried out on an ESCALAB 250 instrument (Thermo Scientific Inc., USA).

**Electrochemical measurements**

All electrochemical tests were conducted on an electrochemical workstation (760e, Shanghai Chenhua Instrument Inc., China). All the measurements were carried out in an H-type cell (30 mL per chamber) with a Nafion 117 membrane. The three-electrode system consists of an Ag/AgCl electrode, a Pt counter electrode, and a working electrode with as-prepared catalysts. The foams of the working electrode were cut into the size of 1.0 cm × 3 cm, and the geometric surface area for electrocatalytic tests is 2 cm^2^ (1.0 cm × 2.0 cm). In each test, 18 mL 50 mM Na_2_SO_4_ served as the electrolyte in both chambers and 10 mM HMF was added to the cathodic part and electrolyzed for 2 h. The scan rate of 10 mV s^-1^ was adopted to collect the CV and LSV curves. Double-layer capacitance was measured by CV in 0.1 M Na_2_SO_4_ under open circuit voltage with different scan rates. EIS analysis was conducted with 5 mV amplitude over a frequency range from 0.1 to 105 Hz. All the experiments were carried out at room temperature.

**Product Analysis**

Liquid samples were collected and diluted with deionized water for analysis with high-performance liquid chromatography (HPLC, LC-16, Shimadzu Co., Japan) equipped with a UV-vis dual wavelength detector (SPD-16). The column (ZORBAX Eclipse Plus C18, 4.6×250 mm, 5 µm) was operated at 35 °C with a binary gradient pumping method of water and CH_3_CN at 0.6 mL min^-1^ flow rate. The CH_3_CN fraction was increased from the initial 15% (v/v) to 60% over the 4-5 min period, then was kept at 60% over the 5-7 min period, and then was decreased to 15% from the 7-8 min period. The UV detector was set at 240 nm. DHMF and HMF were eluted at around 6.9 and 8.4 min, respectively. Two isomers of the HMF dimer, 5,5’-bis(hydroxymethyl)hydrofuroin (denoted as BHH), were eluted at different retention times (6.4 and 7.4 minutes). HMF and electrocatalytic reduced products were identified and quantified by comparison to genuine samples, except for BHH. Since a commercial sample was not available for BHHs, the calibration was estimated as twice that of BHMF.^[1, 2]^ A similar procedure was used for quantifying other electrocatalytic reductive products.

HMF reduction products were also determined by liquid chromatography-tandem mass (LC-MS, Triple TOF 5600 Instrument, AB SCIEX Co., USA) using electrospray ionization (ESI) in MS and MS/MS modes. The measurements were conducted in a positive ion mode (interface capillary voltage 4500 V), and the range of mass scanning m/z was 50-1000 Da.

The conversion (%) of HMF, and the selectivity of DHMF (%) have been calculated by following equations a) and b) below, respectively.

Conversion (%) = (mole of substrate consumed) / (mole of initial substrate) ×100% a)

Selectivity (%) = (mole of product formed) / (mole of substrate consumed) ×100% b)

**Density functional theory calculations**

All calculations were performed using the Vienna ab initio simulation package (VASP). Electron-ion interactions were described via the projector-augmented wave (PAW) method. The expansion of wave functions was carried out in a plane wave basis, with an energy cutoff set at 400 eV. Electron exchange-related energies were calculated employing the generalized gradient approximation (GGA) of Perdew-Burke-Ernzerhof (PBE). Grimme's DFT-D3 dispersion correction method was utilized to address the dispersion interactions within the system. Lattice atoms, not fixed to the bulk phase, were allowed to relax until the force was below 0.03 eV/Å. For an optimal balance of calculation accuracy and efficiency, the gamma point served as the sampling mesh in the Brillouin zone for k-points. The adsorption energy of HMF (Eads) was calculated as follows:

E_ads_= E_adsorbate-surface_-E_gas_-E_slab_ (1)

Where E_adsorbate_, E_gas_, and E_slab_ represent the energy of the adsorbate-surface coupler, the gaseous adsorbate molecule, and the clean surface, respectively. The climbing image nudged elastic band (CINEB) method and the dimer method were employed to identify the transition state. The CINEB method facilitated the initial convergence of the transition state when the maximum force was below 0.1 eV/Å. Subsequently, the dimer method was utilized to further refine the position of the transition state. Convergence at the saddle point was achieved when interatomic forces fell below 0.03 eV/Å.**Supplementary Figures**

**
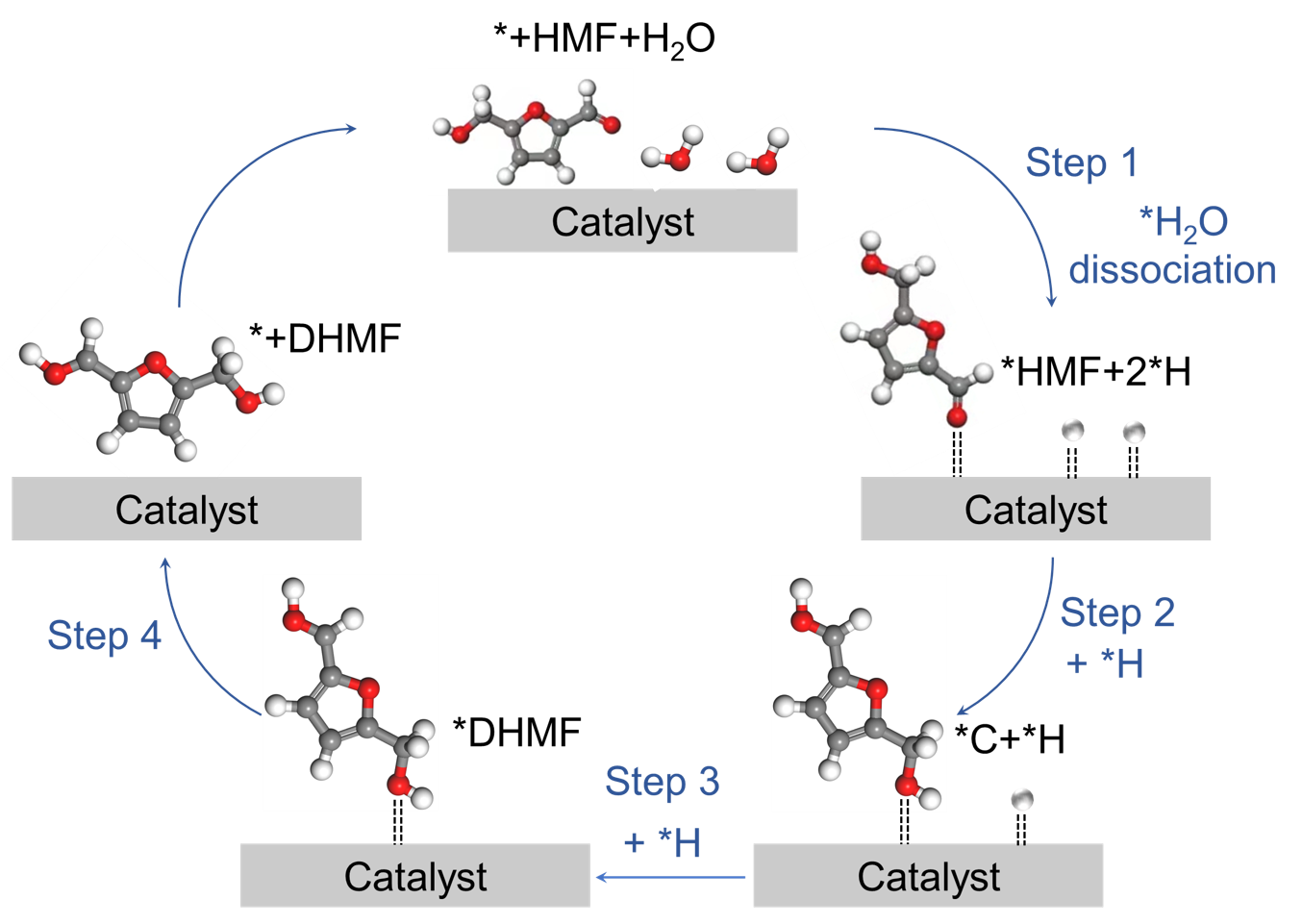
**

Figure S1. Schematic illustration of the HAT reaction mechanism.


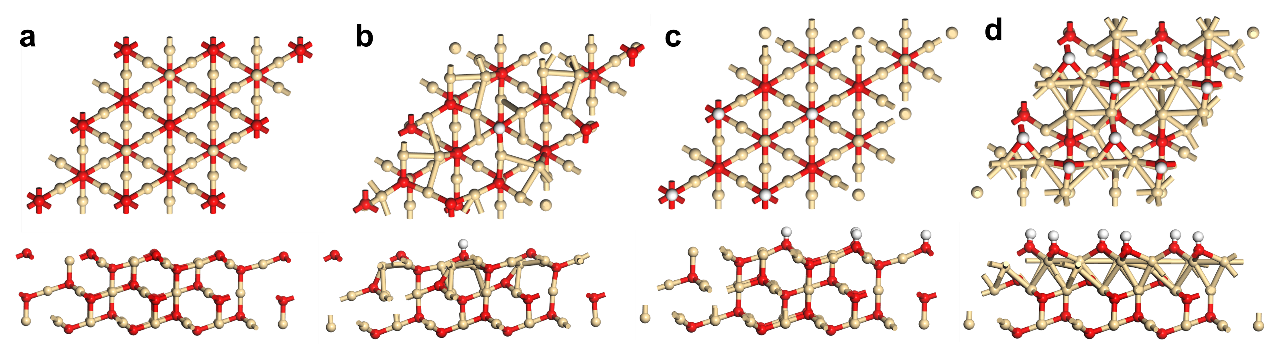


Figure S2. Optimized configurations on Cu_2_O (111) surface under 0 *H nm^-2^ (a), 1 *H nm^-2^ (b), 4 *H nm^-2^ (c), and 8 *H nm^-2^ (d) coverage conditions.

**
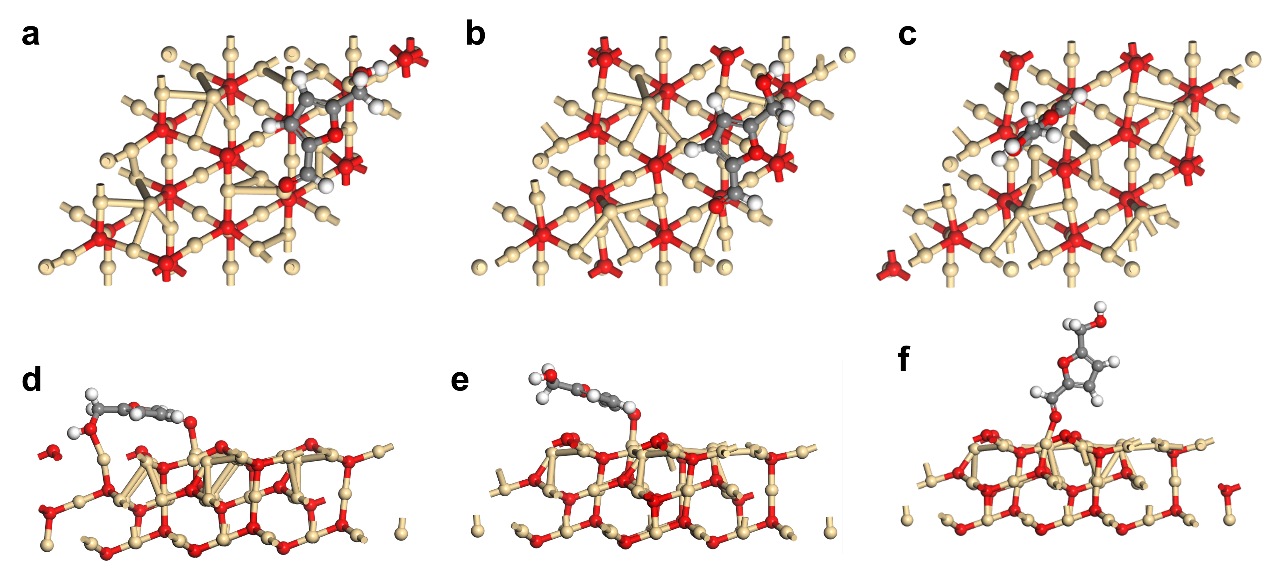
**

Figure S3. Optimized adsorption configurations of HMF on Cu_2_O Surface: (a, d) flat-Lying, (b, e) tilted, and (c, f) vertical Orientations.

**
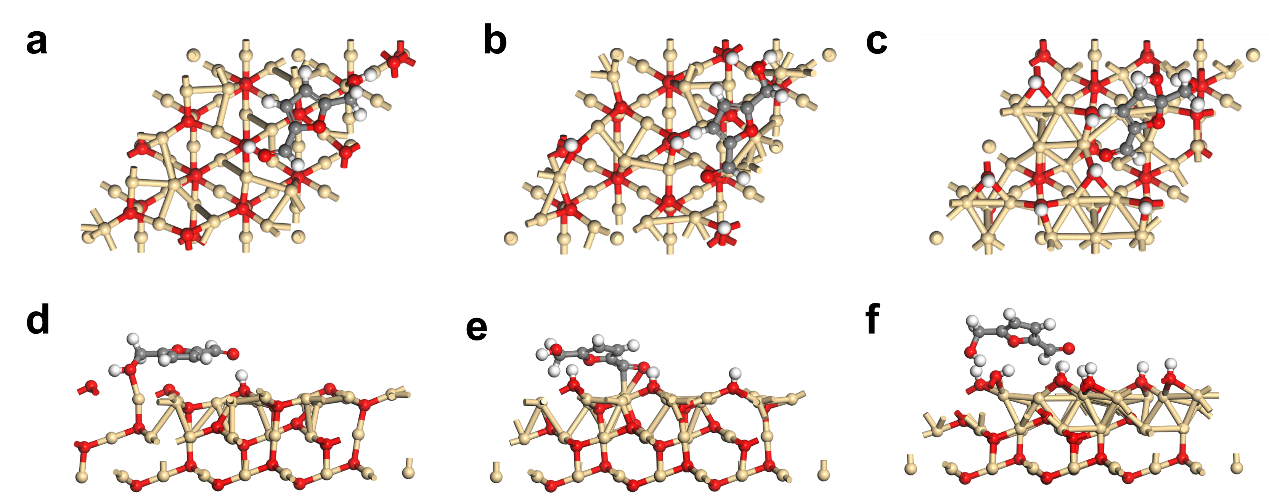
**

Figure S4. Optimized adsorption configurations of HMF on Cu_2_O Surface under 1 *H nm^-2^ (a, d), 4 *H nm^-2^ (b, e), and 8 *H nm^-2^ (c, f) coverage conditions.


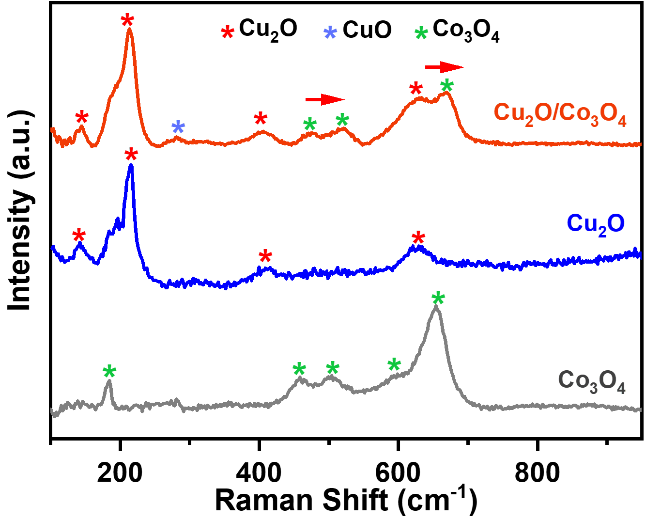


Figure S5. Raman spectra of Co_3_O_4_, Cu_2_O, and Cu_2_O/Co_3_O_4_.

**
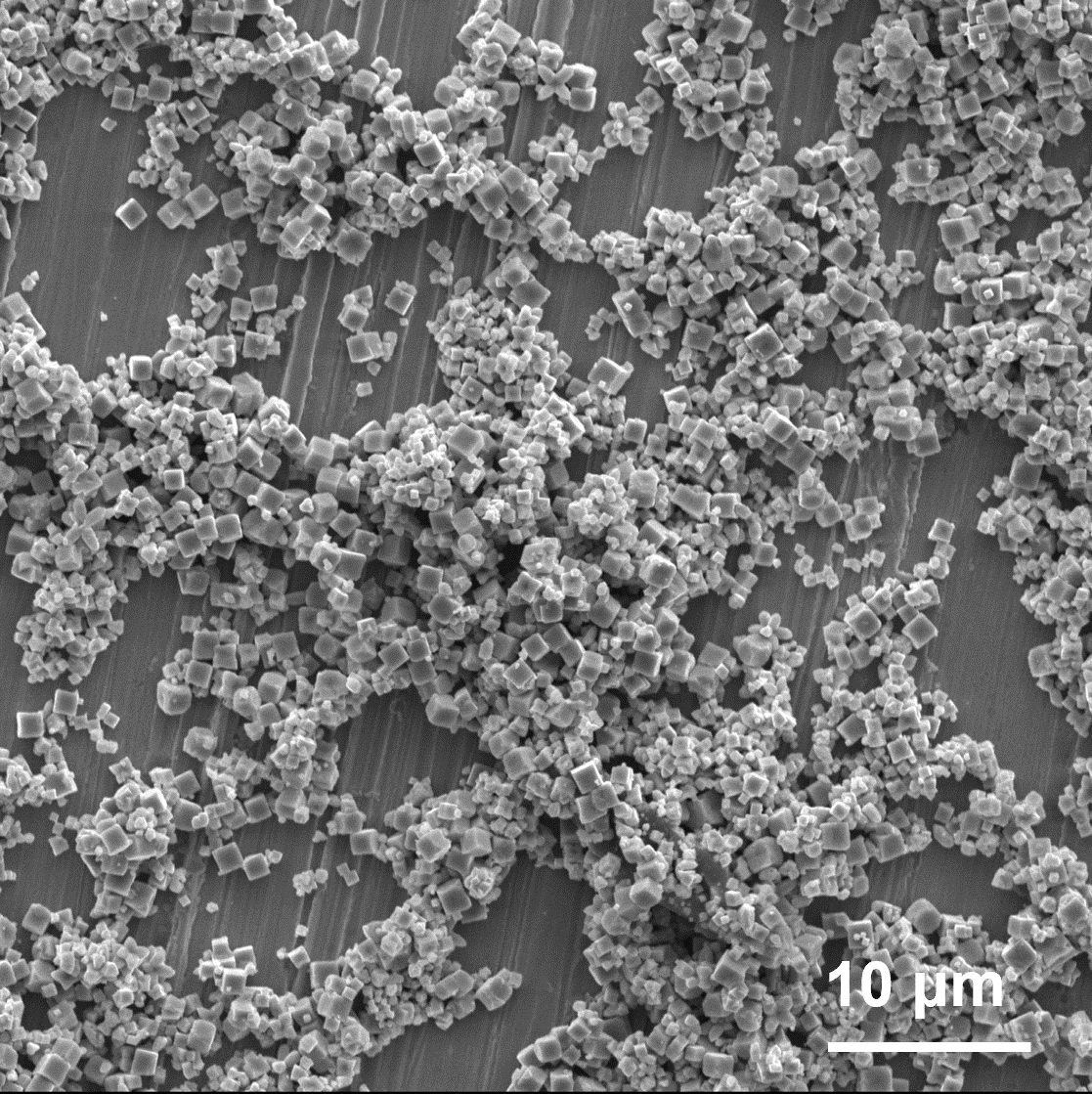
**

**Figure S6**. SEM images of Cu_2_O.

**
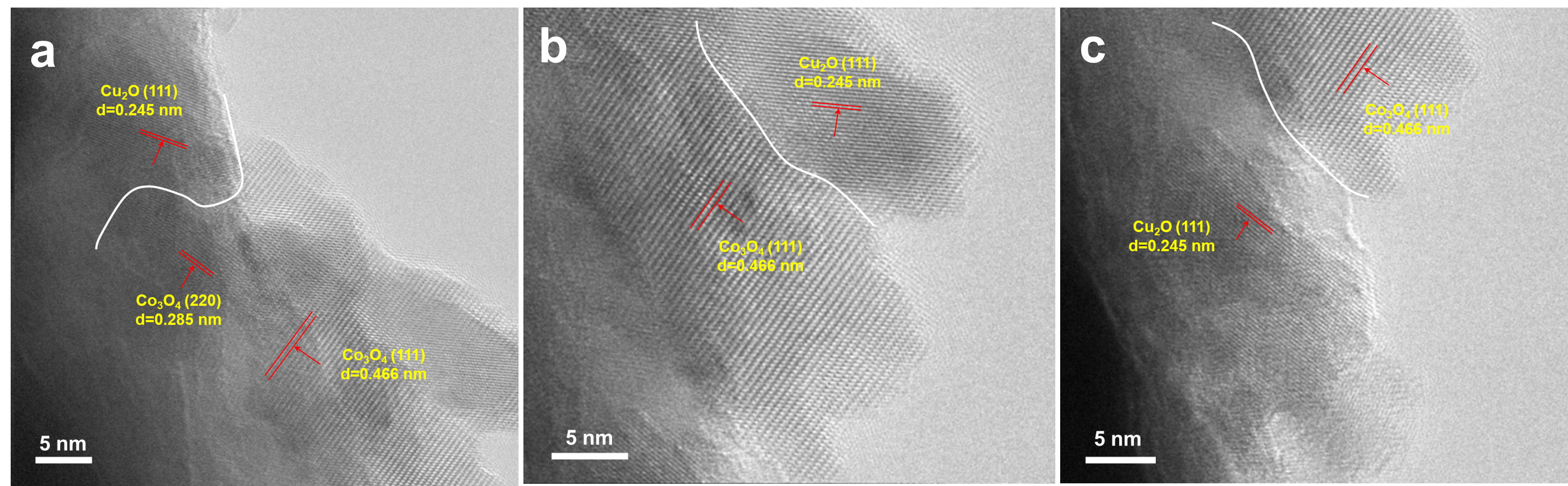
**

**Figure S7**. HRTEM images of Cu_2_O/Co_3_O_4_ in different fields of view.

**
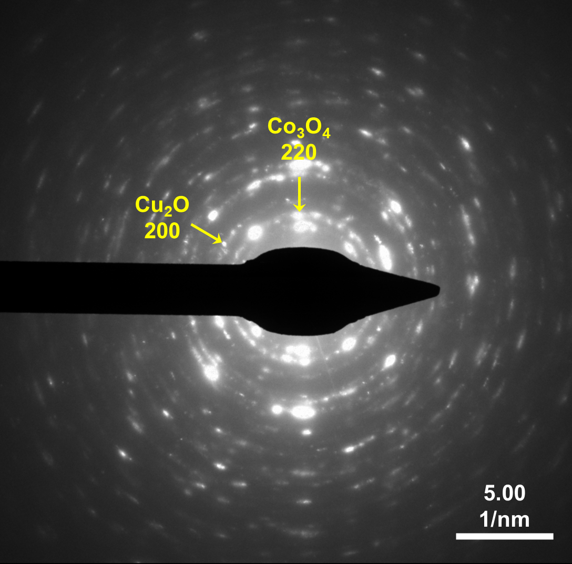
**

**Figure S8**. Selected-area electron diffraction (SAED) patterns of Cu_2_O/Co_3_O_4_.


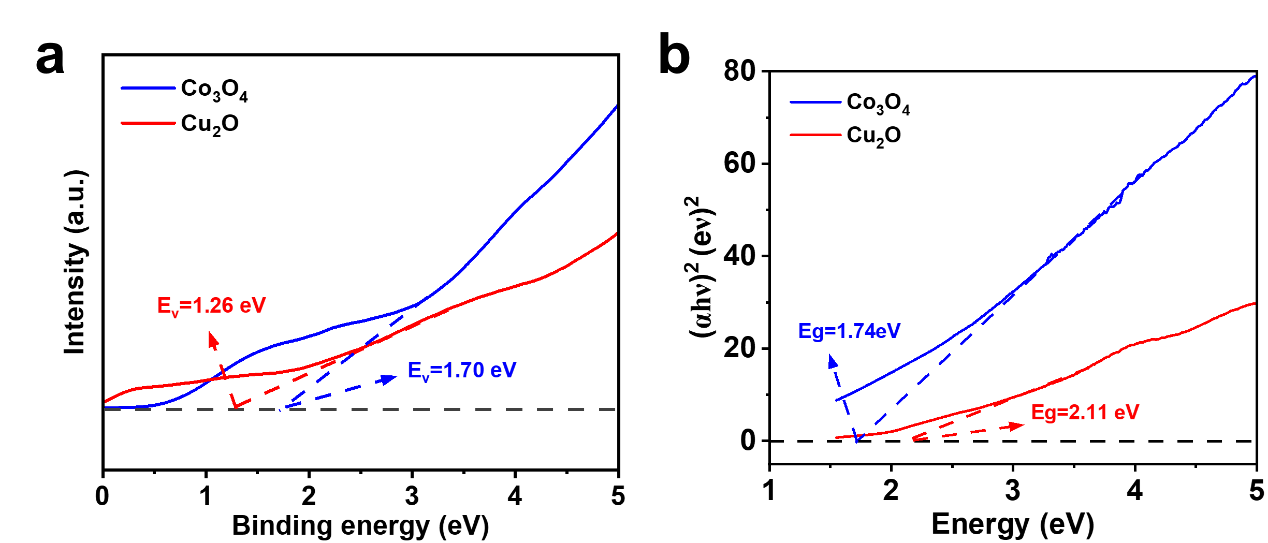


Figure S9. (a) UPS spectra of Co_3_O_4_ and Cu_2_O; (b) UV-vis spectra of Co_3_O_4_ and Cu_2_O.


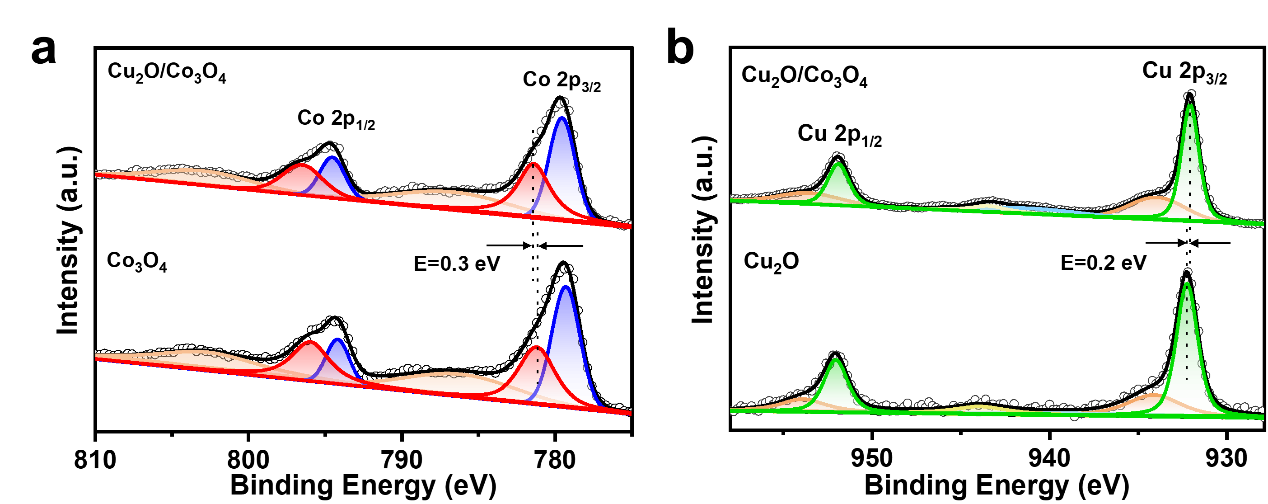


Figure S10. (a) XPS spectra of Co 2p; (b) XPS spectra of Cu 2p.


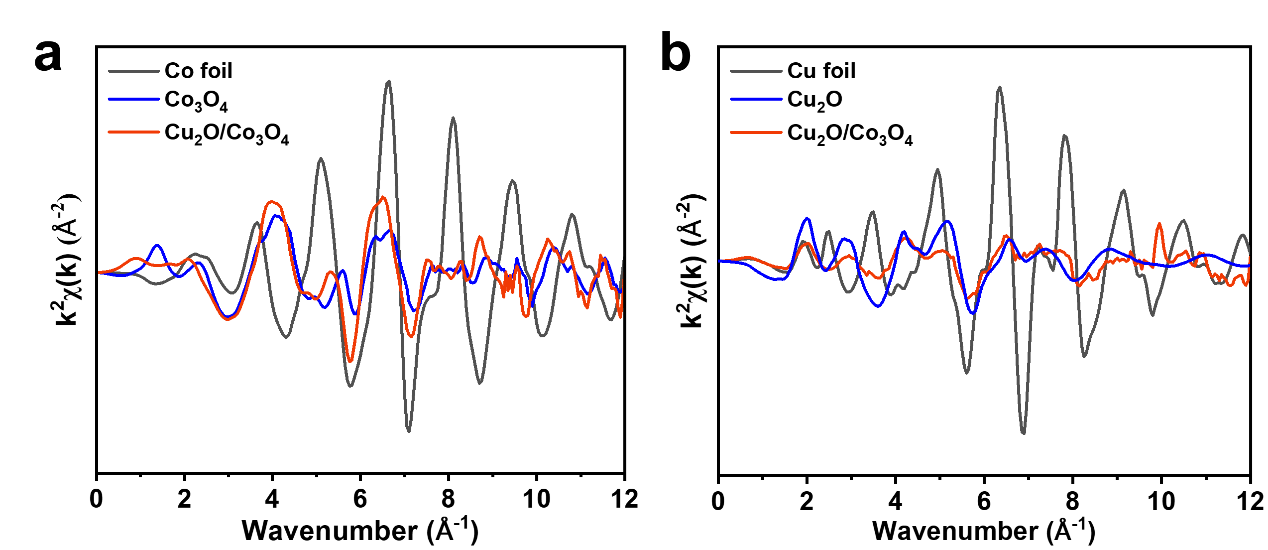


**Figure S11.** (a) k2χ(k) functions of Co K-edge EXAFS spectra of Co_3_O_4_ and Cu_2_O/Co_3_O_4_ catalysts; (b) k2χ(k) functions of Cu K-edge EXAFS spectra of Cu_2_O and Cu_2_O/Co_3_O_4_ catalysts.


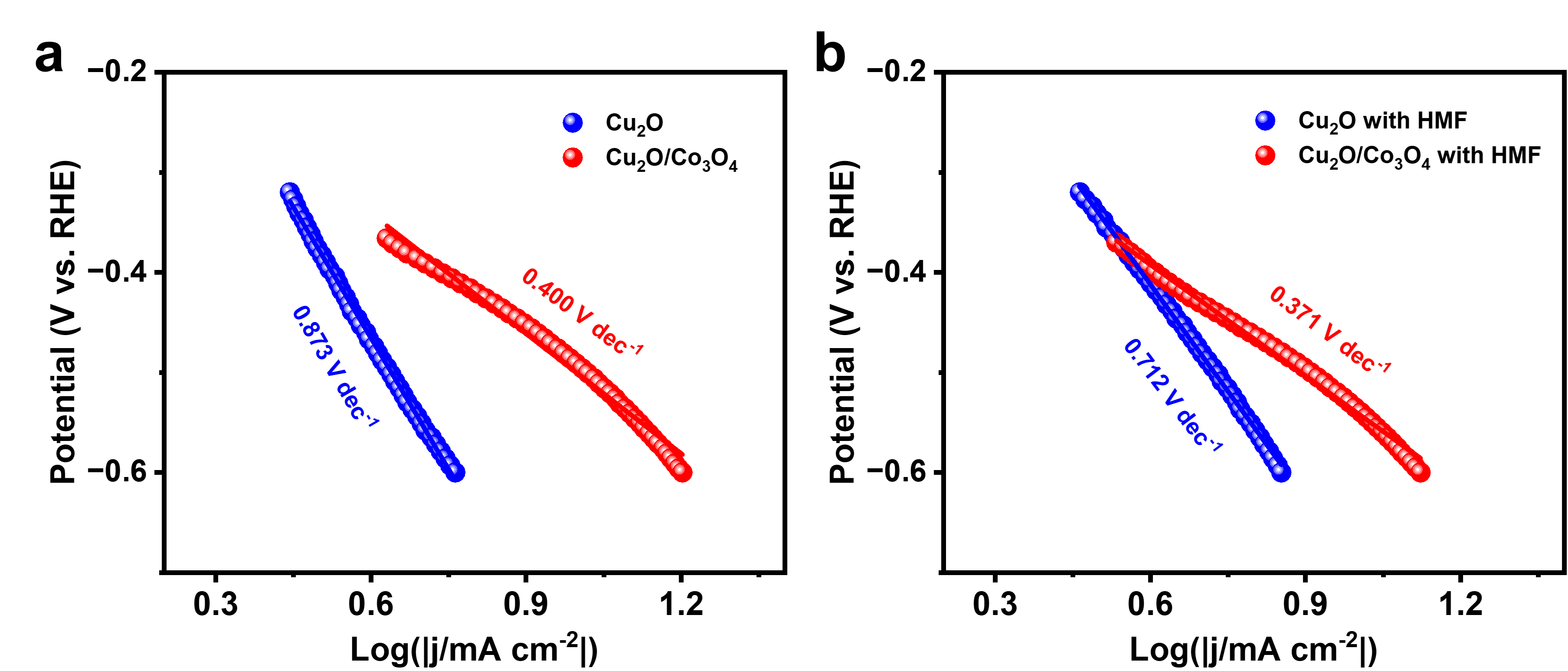


**Figure S12.** Tafel slope of Cu_2_O and Cu_2_O/Co_3_O_4_ in 0.1 M Na_2_SO_4_ electrolyte without (a) and with (b) the addition of 10 mM HMF.


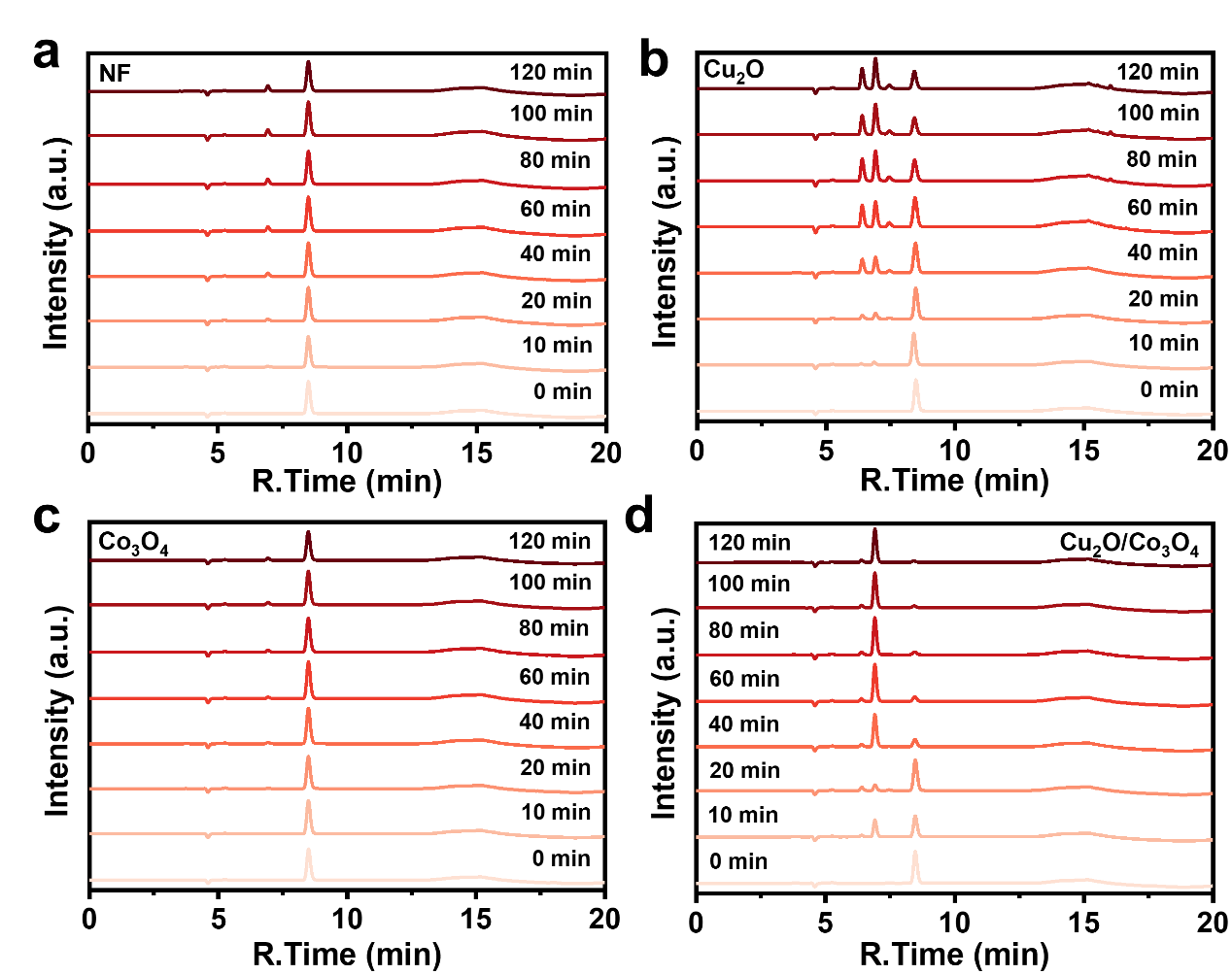


Figure S13. Liquid chromatogram of reactant and products over Ni Foam, Cu_2_O, Co_3_O_4_, Cu_2_O/Co_3_O_4_ in 0.1 M Na_2_SO_4_ with 10 mM HMF at -0.6 V vs. RHE.


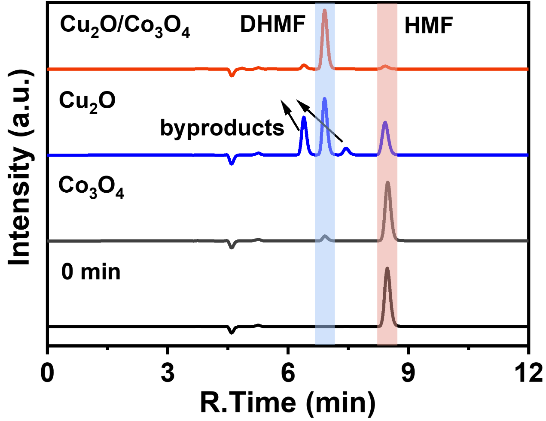


Figure S14. Liquid chromatogram of reactant and products over Cu_2_O/Co_3_O_4_, Cu_2_O, Co_3_O_4_ in 0.1 M Na_2_SO_4_ with 10 mM HMF at -0.6 V vs. RHE.


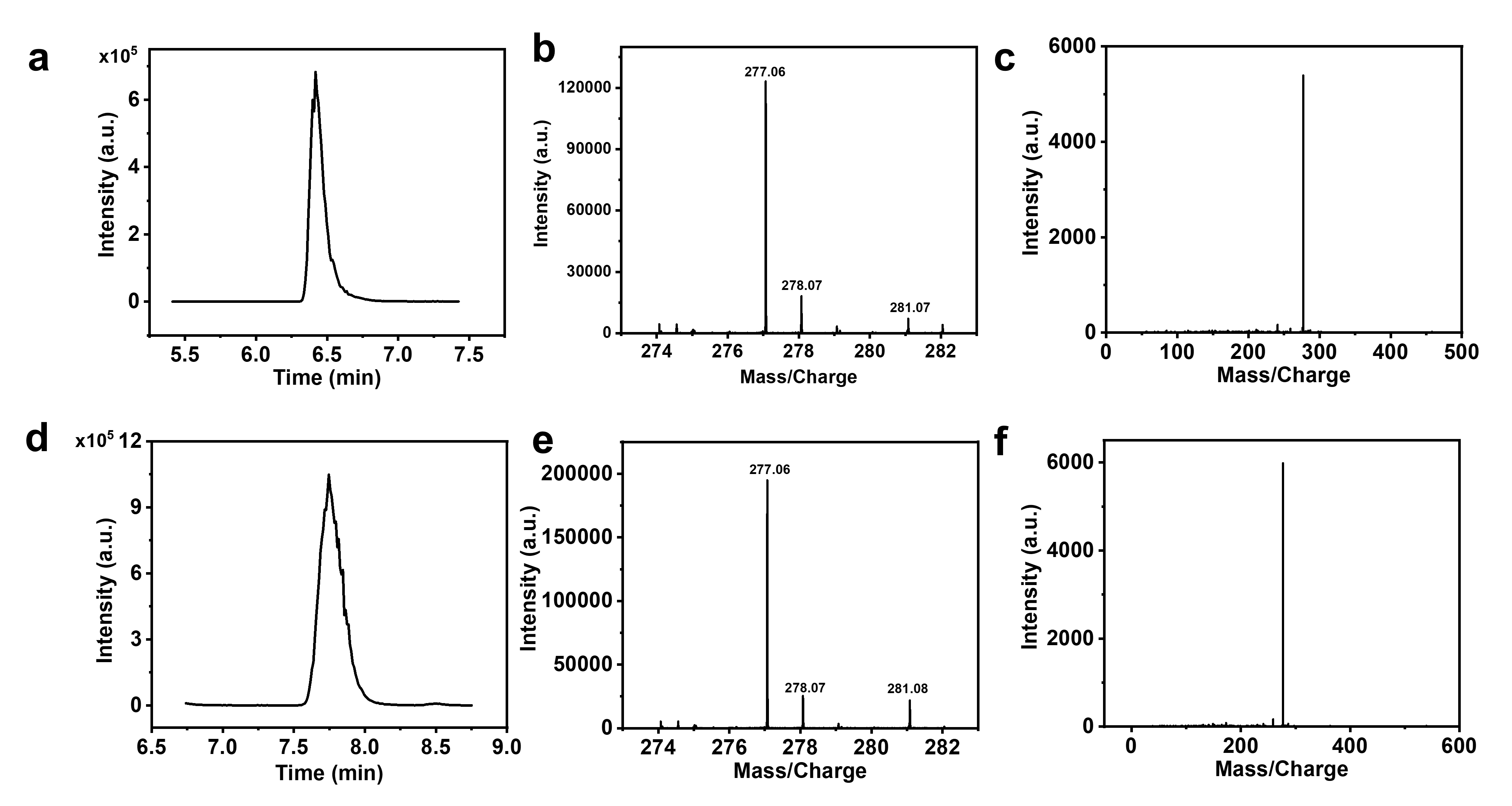


Figure S15. LC-MS spectra of BHH.


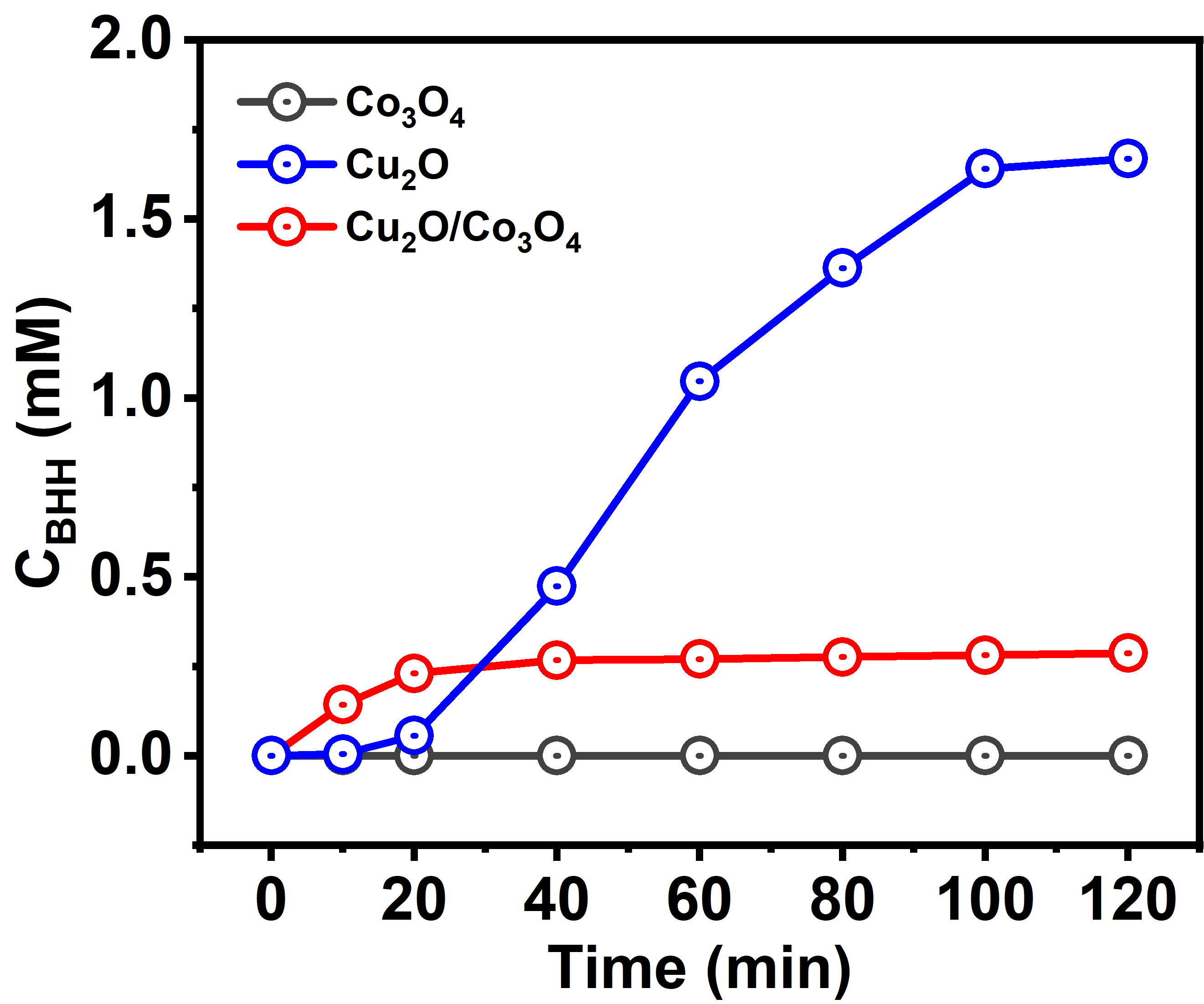


Figure S16. Time-dependent concentration changes of BHH on Cu_2_O/Co_3_O_4_, Cu_2_O, and Co_3_O_4_.


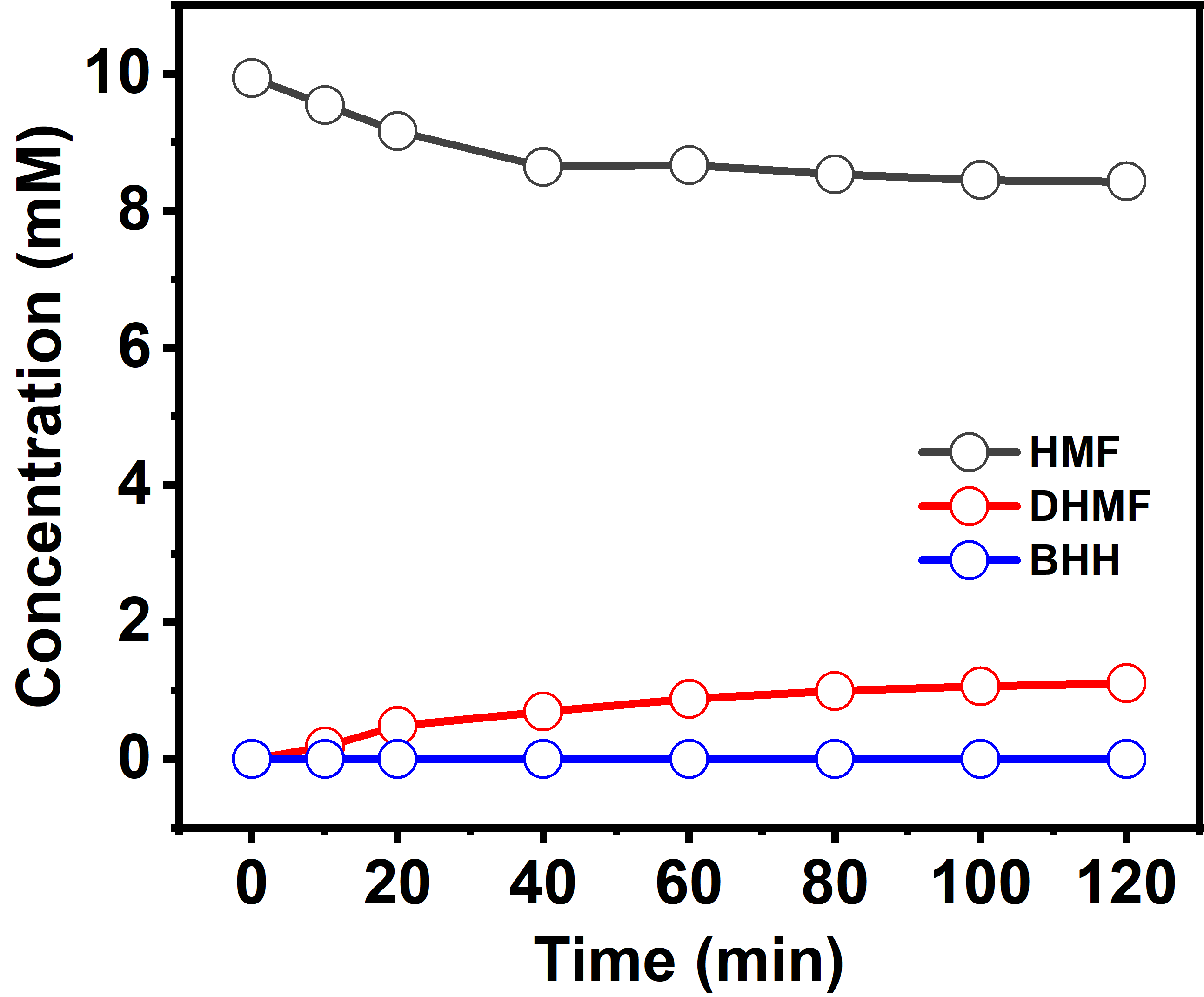


Figure S17. Time-dependent concentration changes of HMF, DHMF, and BHH on Ni Foam.


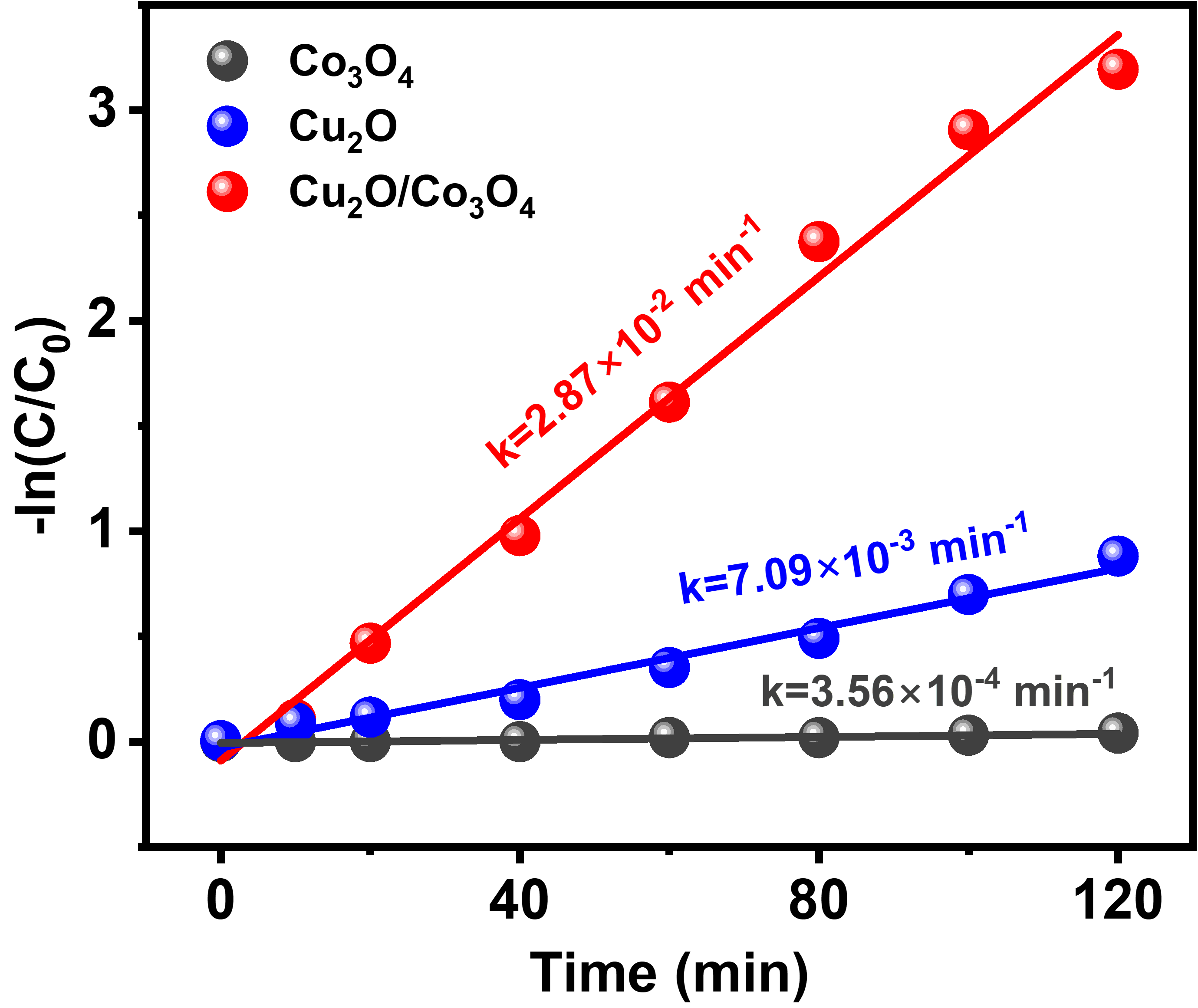


Figure S18. Kinetic fitting curves for the reduction of 5-HMF to DHMF by Cu_2_O/Co_3_O_4_, Cu_2_O, and Co_3_O_4_.


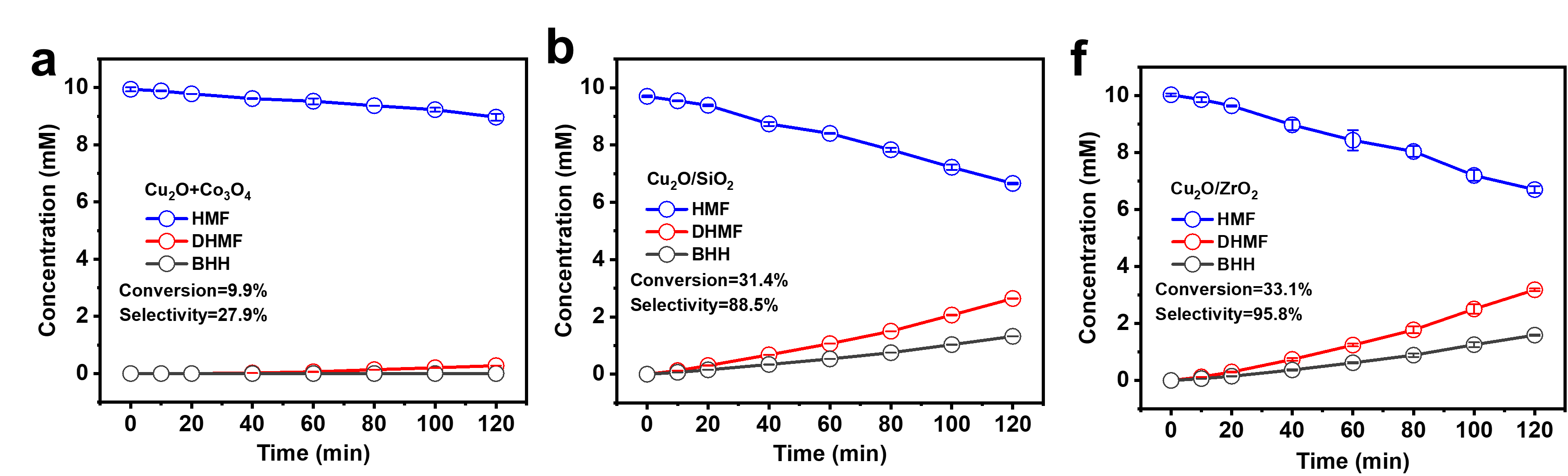


Figure S19. Concentration evolution of 5-HMF, DHMF and BHH during 5-HMF reduction on (a) physical mixture of Cu_2_O + Co_3_O_4_, (b) Cu_2_O/SiO_2_ and (c) Cu_2_O/ZrO_2_.


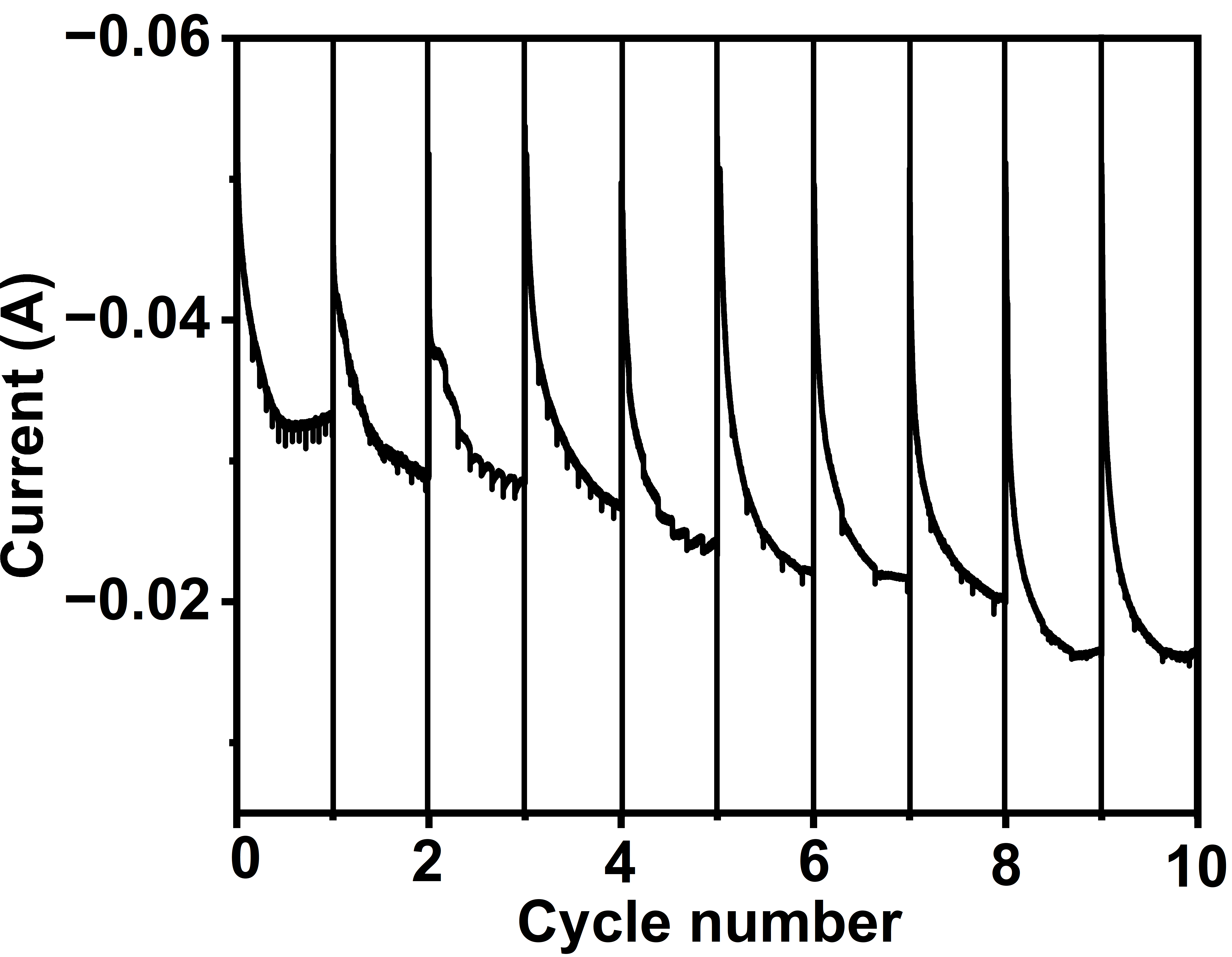


Figure S20. I-T curve of Cu_2_O/Co_3_O_4_ catalyst during 10 cycles.

**
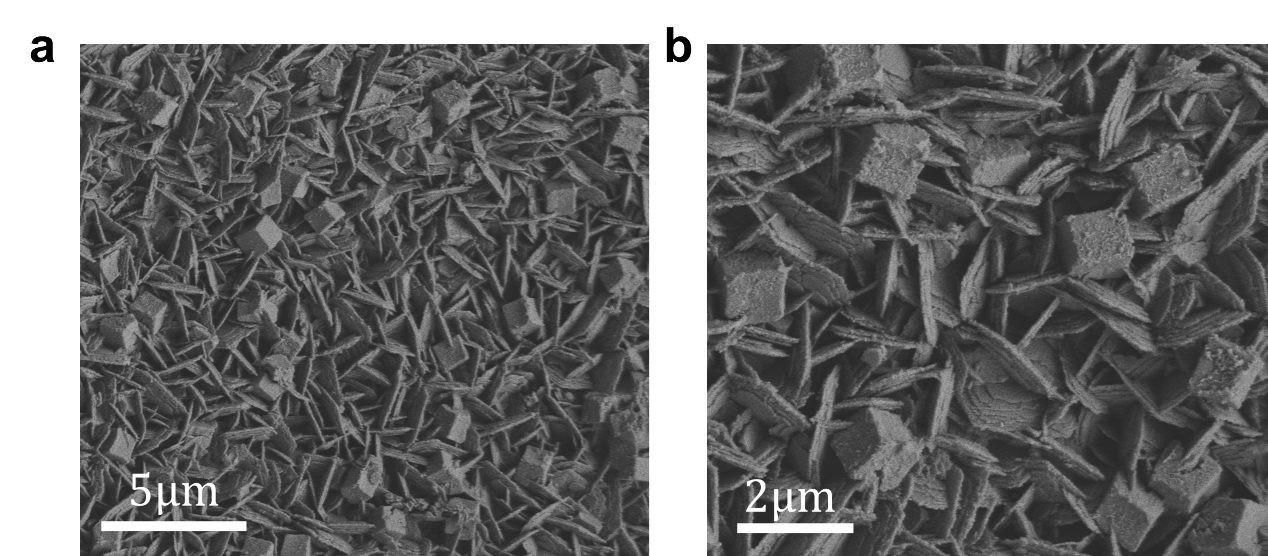
**

Figure S21. (a, b) SEM of Cu_2_O/Co_3_O_4_ after 10 cycles..
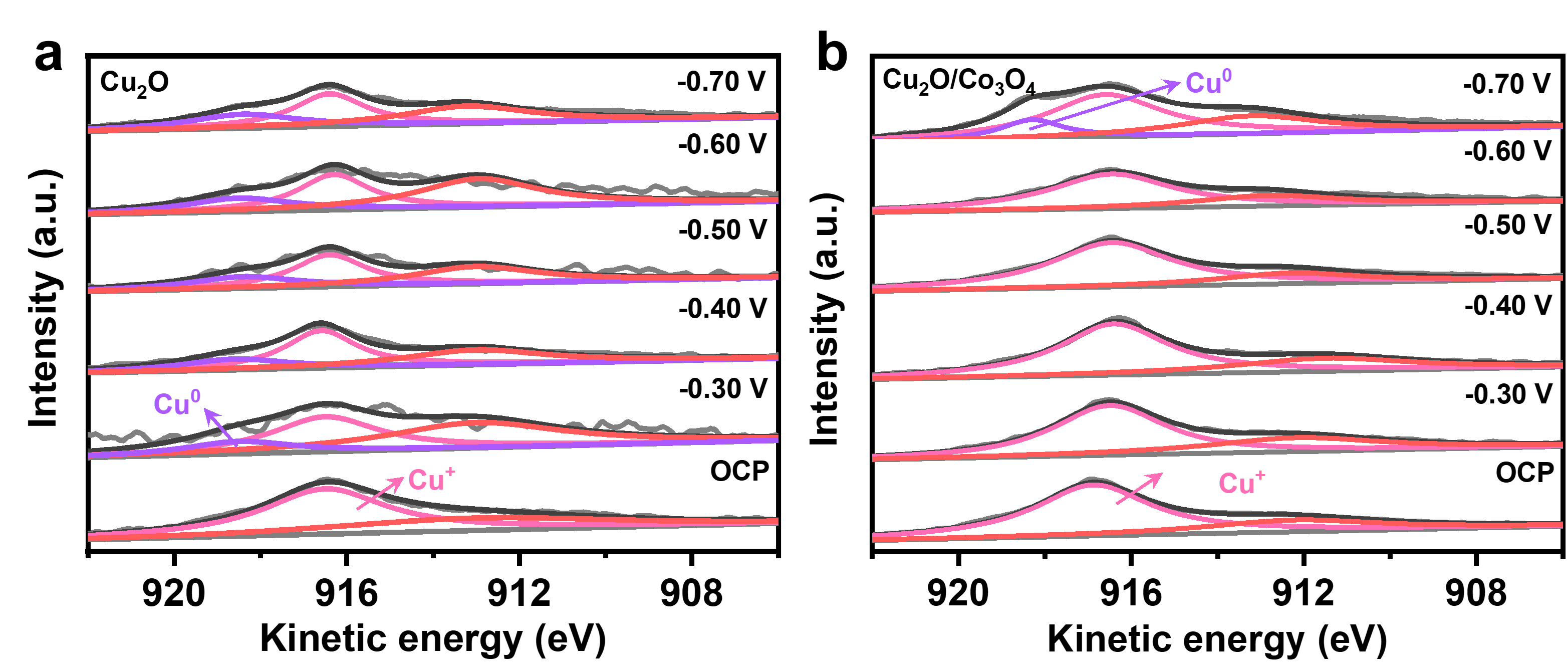


**Figure S22.** Quasi-in-situ XPS data acquired at open-circuit and cathodic potentials for Cu_2_O (a) and Cu_2_O/Co_3_O_4_ (b).

Figure S23. Time-Dependent XPS analysis of Cu species


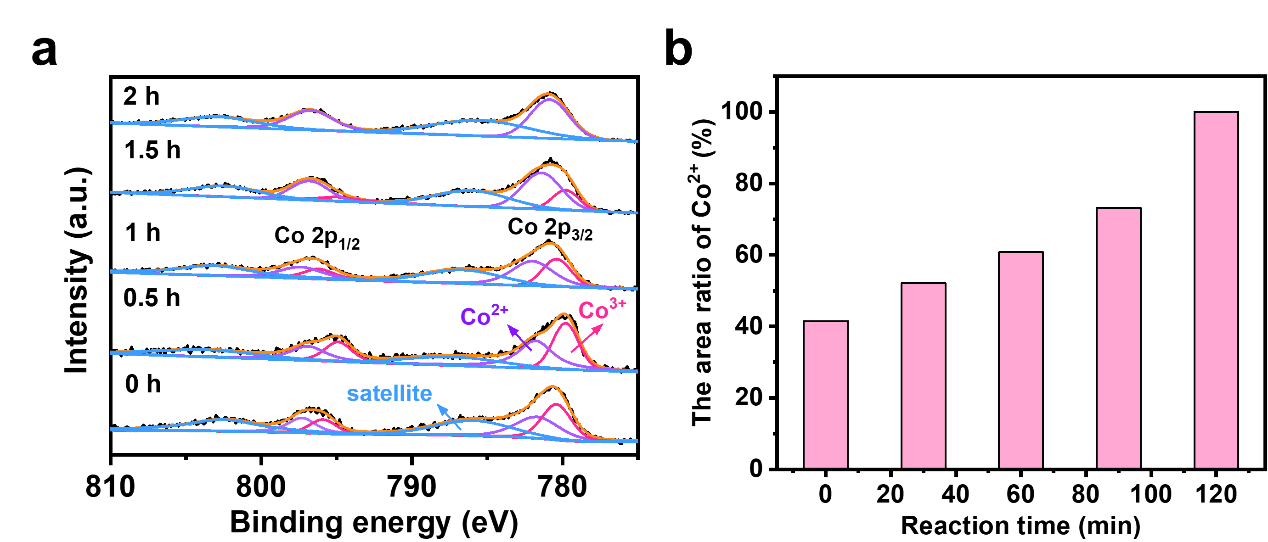


**Figure S24.** (a) Time-Dependent XPS analysis of Co species; (b) Evolution of Co²⁺ species ratio over time.

Figure S25. H_2_ TPD of Cu_2_O/Co_3_O_4_, Cu_2_O, and Co_3_O_4_.

The reducibility of Cu_2_O with and without support was explored with H_2_-TPR. Co_3_O_4_ shows a broad reduction peak at 334 ℃, which corresponds to the reduction of Co^3+^ to Co^2+^ and subsequent Co^2+^ to metallic Co.^[3, 4]^ Synthesized Cu_2_O showed two reduction peaks at 209 and 274 ℃, corresponding with the reduction of Cu^2+^ to Cu^+^ and the reduction of Cu^+^ to Cu. ^[5, 6, 7]^By contrast, there are four peaks in Cu_2_O/Co_3_O_4_, at 195 ℃, 334 ℃, 373 ℃ and 401 ℃, respectively. Comparing these peaks with those of Co_3_O_4_ and Cu_2_O and considering the peak shape, we attributed the peaks at 195 ℃ to Cu^2+^$\boldsymbol{\to}$Cu^+^, and the peaks at 334 ℃ and 401 ℃ to Co^3+^$\boldsymbol{\to}$Co^2+^ and Co^2+^$\boldsymbol{\to}$Co^+^, while the peak at 373℃ should be the reduction of Cu^+^ to Cu. These results suggested that the Co_3_O_4_ support stabilized the Cu(I) in Cu_2_O, which may contribute to the enhancement in ECH selectivity.

**
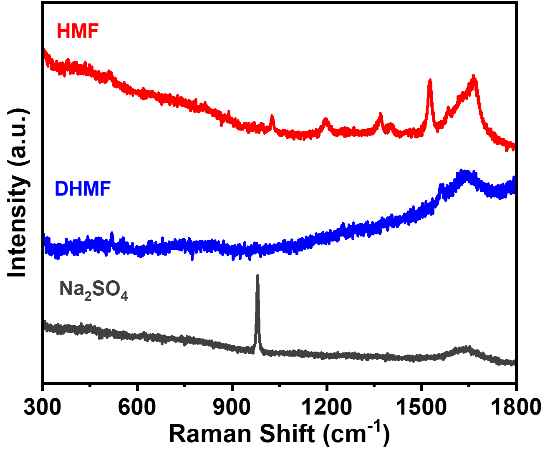
**

Figure S26. Raman spectra of Na_2_SO_4_, DHMF, HMF.


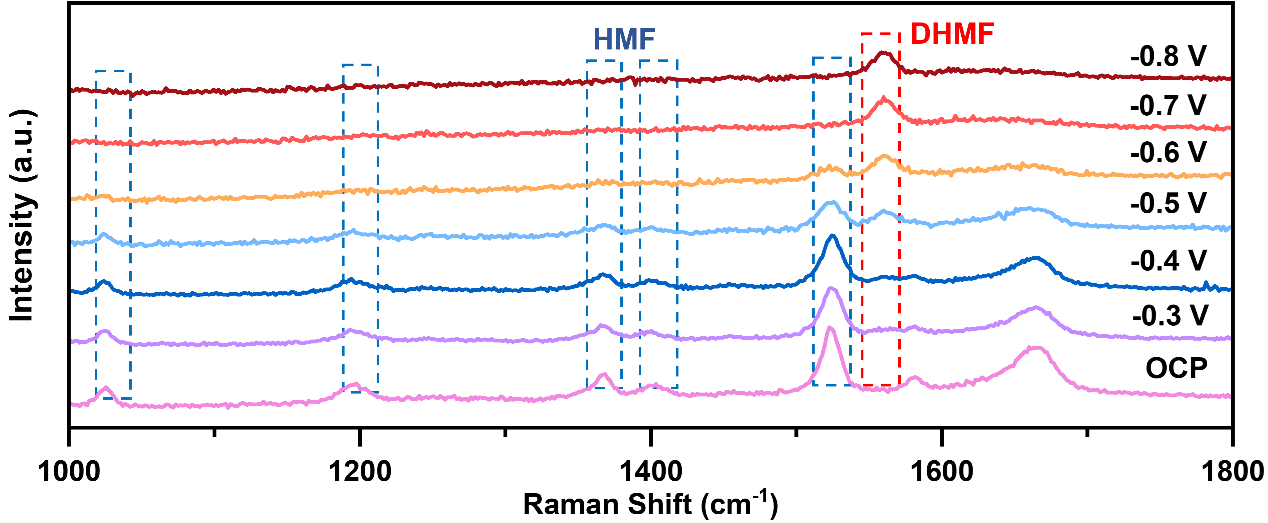


Figure S27. In situ Raman spectroscopy of Cu_2_O/Co_3_O_4_ under different voltages in the 0.1 M Na_2_SO_4_ electrolyte with 50 mM HMF.


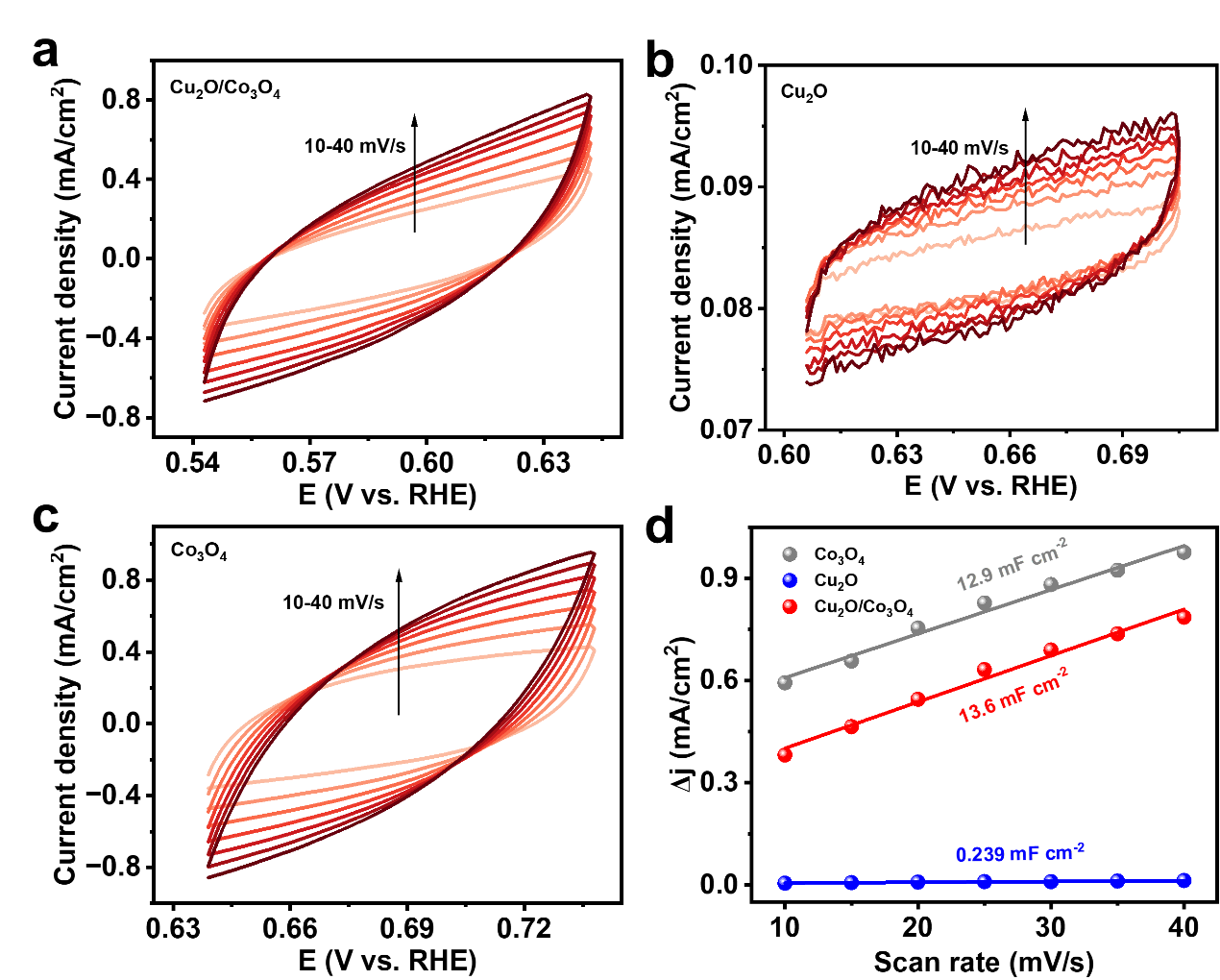


Figure S28. CV curves of (a) Cu_2_O/Co_3_O_4_, (b) Cu_2_O, (c) Co_3_O_4_ in non-faradic regions with different scan rates; (d) double-layer capacitance of Cu_2_O/Co_3_O_4_, Cu_2_O, and Co_3_O_4_.


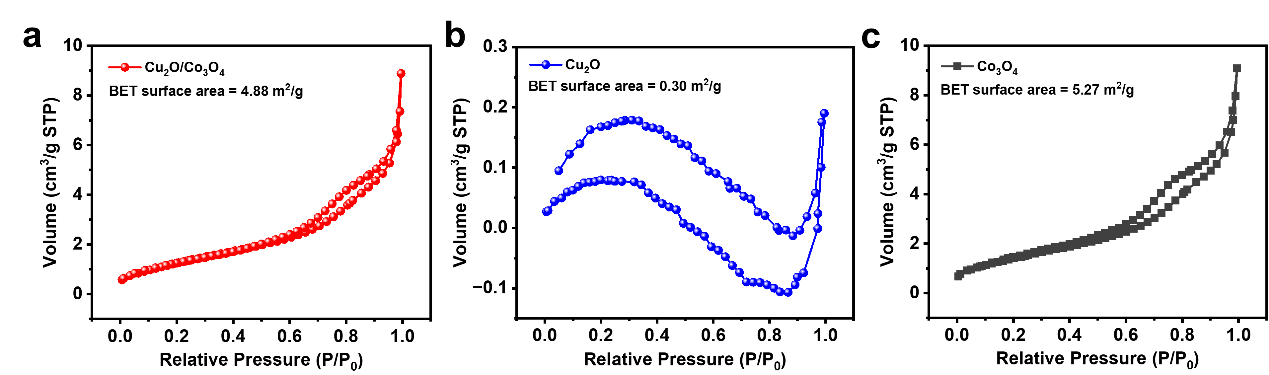


Figure S29. Nitrogen adsorption-desorption isotherms of (a) Cu_2_O/Co_3_O_4_ (b) Cu_2_O and (c) Co_3_O_4_.

**
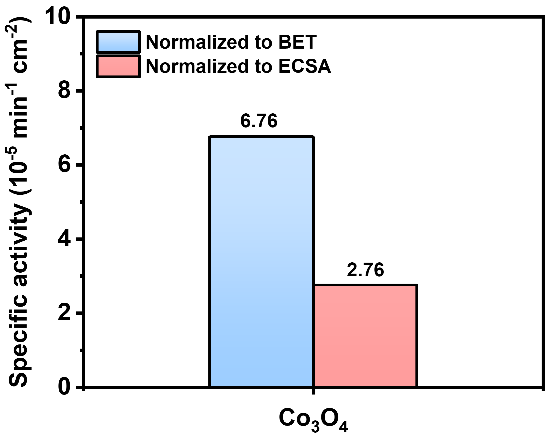
**

Figure S30. Normalized rate constants for Co_3_O_4_, relative to its respective BET and ECSA.


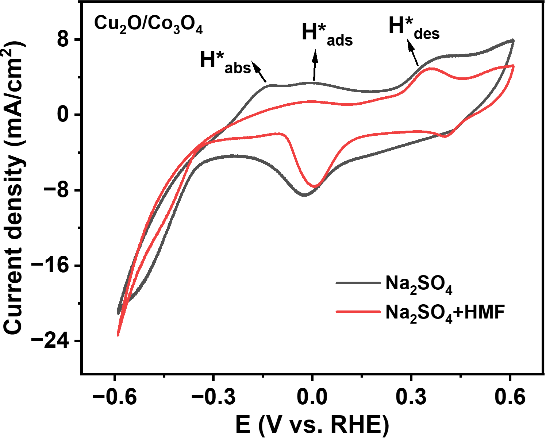


Figure S31. CV curves of Cu_2_O/Co_3_O_4_ in 0.1 M Na_2_SO_4_ with and without 10 mM HMF with a scan rate of 10 mV·s^-1^.


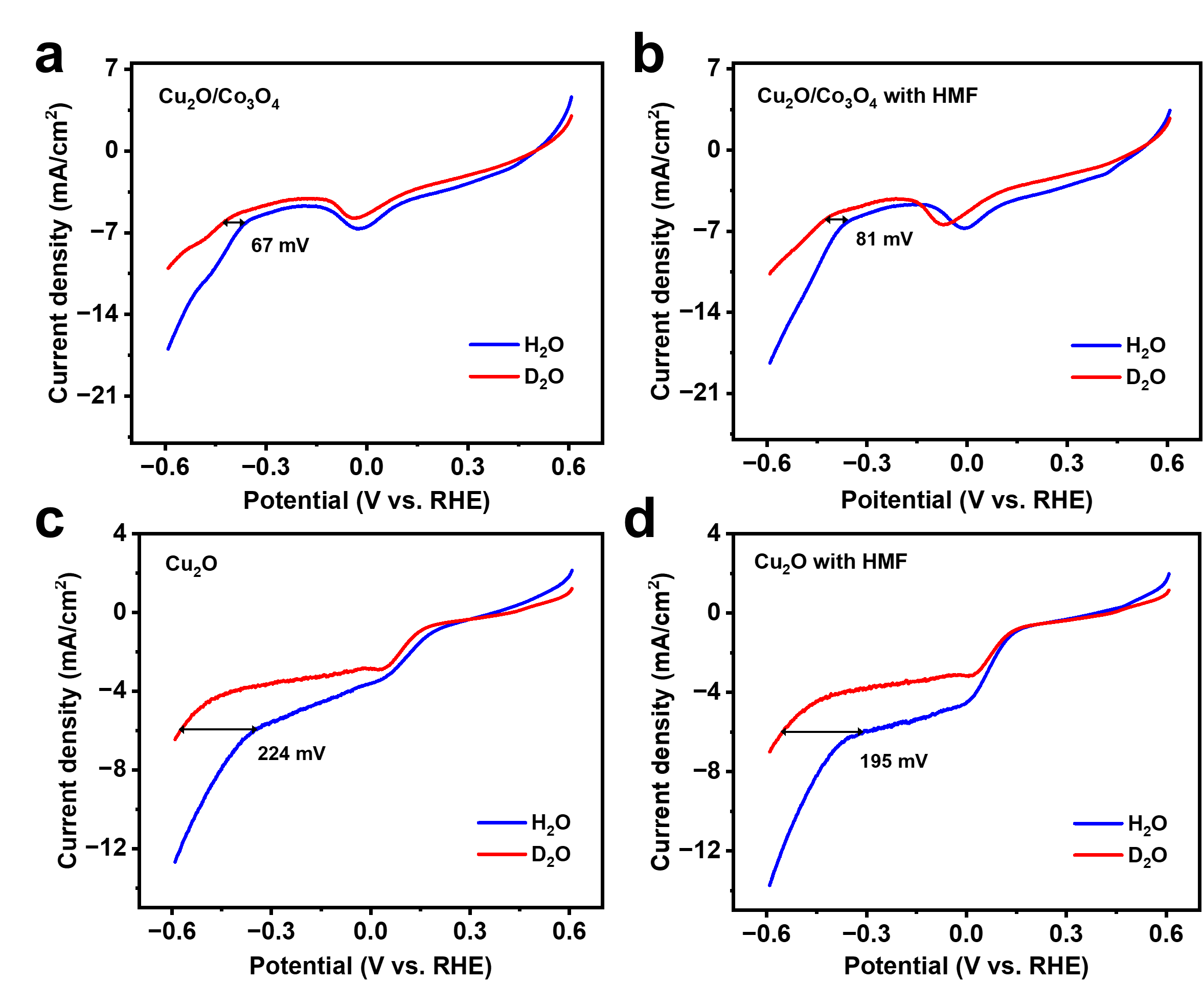


**Figure S32.** Comparison of LSV curves on Cu_2_O/Co_3_O_4_ (a, b) and Cu_2_O (c, d) in pD 7.0 electrolyte without and with the addition of 10 mM HMF.


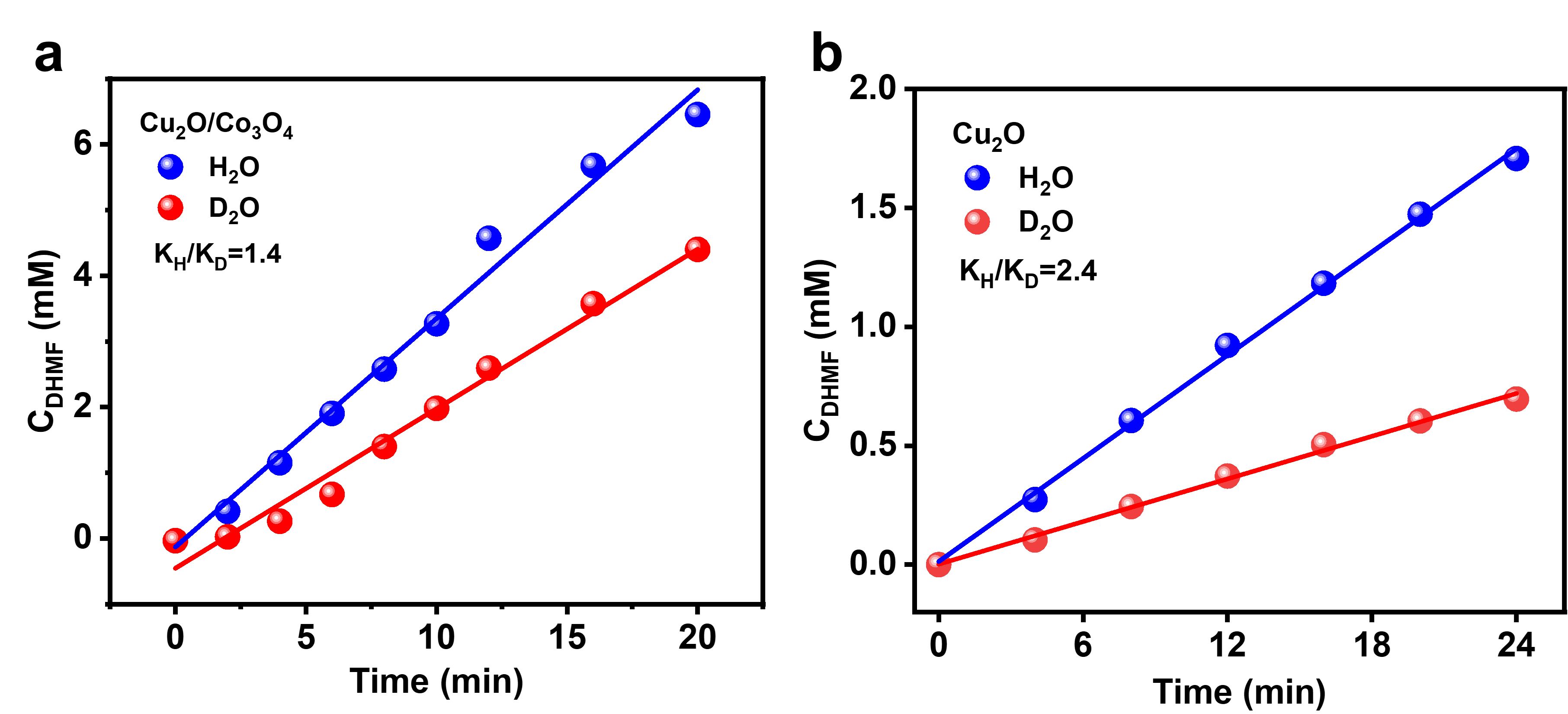


**Figure S33.** The KIE value of Cu_2_O/Co_3_O_4_ (a) and Cu_2_O (b).


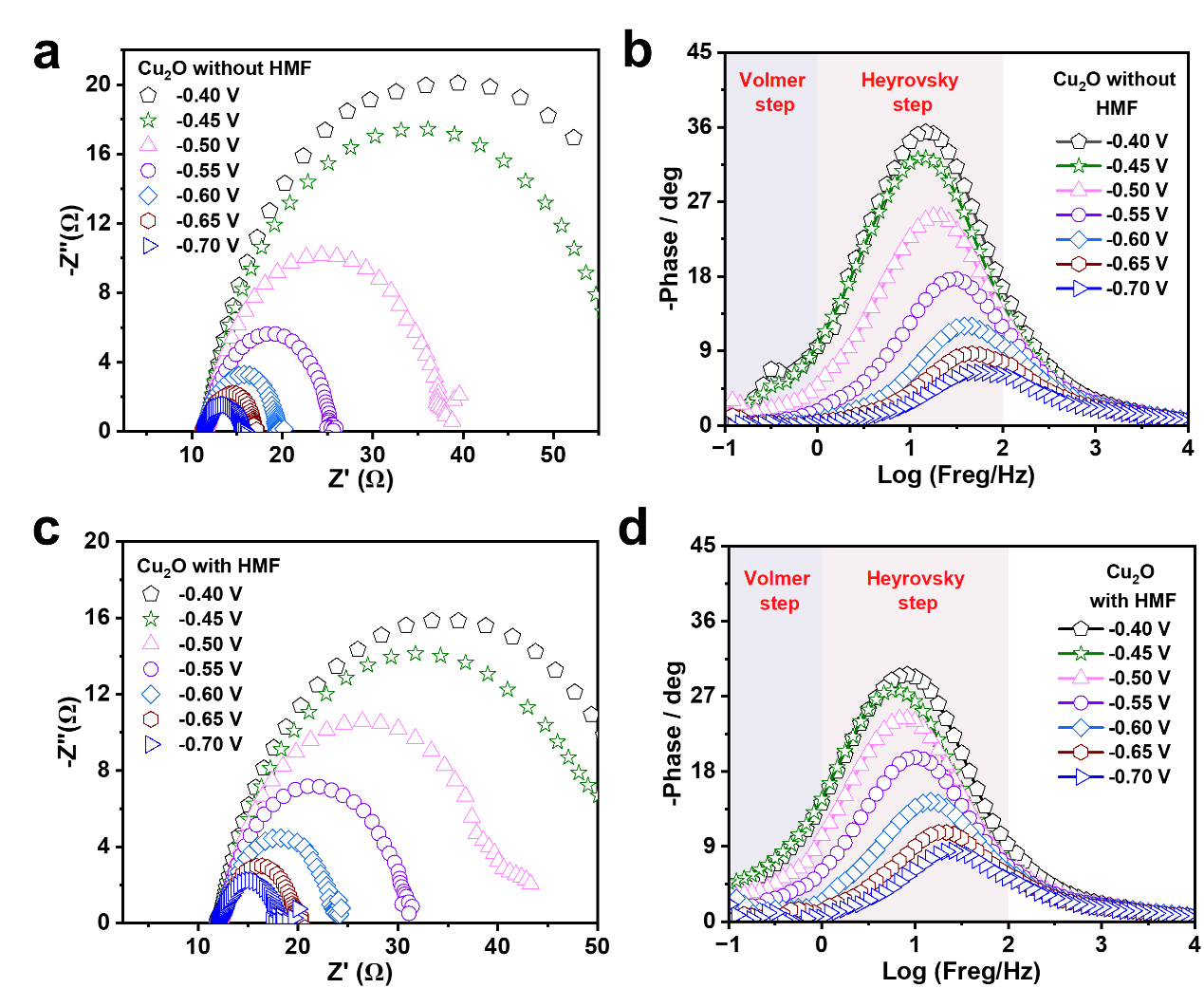


Figure S34. Nyquist and Bode plots for Cu_2_O in (a, b) 0.1 M Na_2_SO_4_ and (c, d) 0.1 M Na_2_SO_4_ with 10 mM HMF.


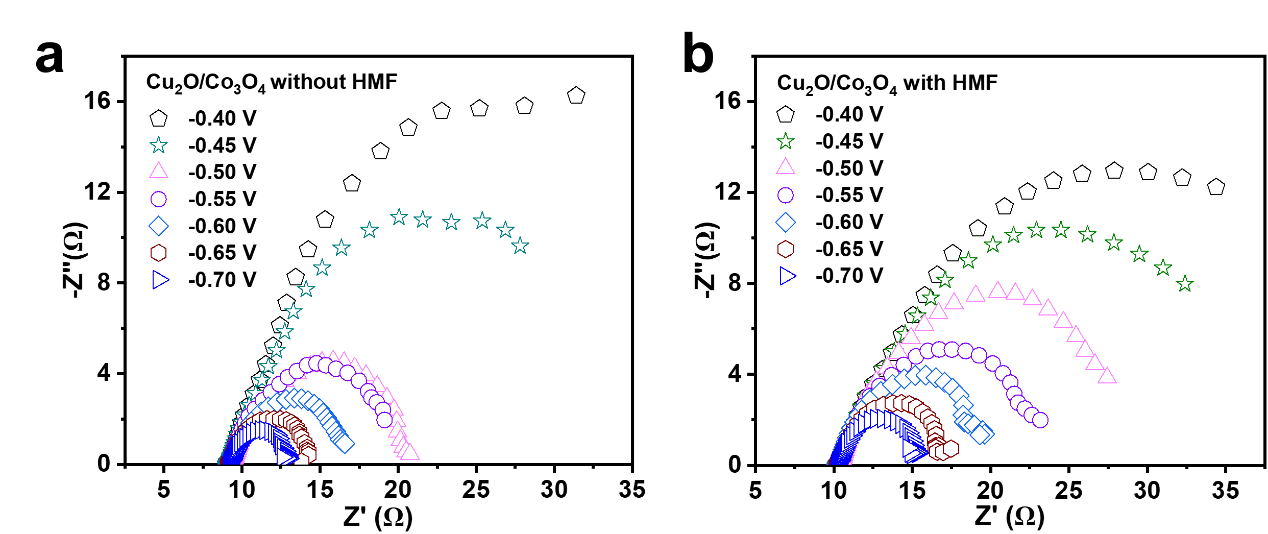


Figure S35. Nyquist plots for Cu_2_O/Co_3_O_4_ in (a) 0.1 M Na_2_SO_4_ and (b) 0.1 M Na_2_SO_4_ with 10 mM HMF.


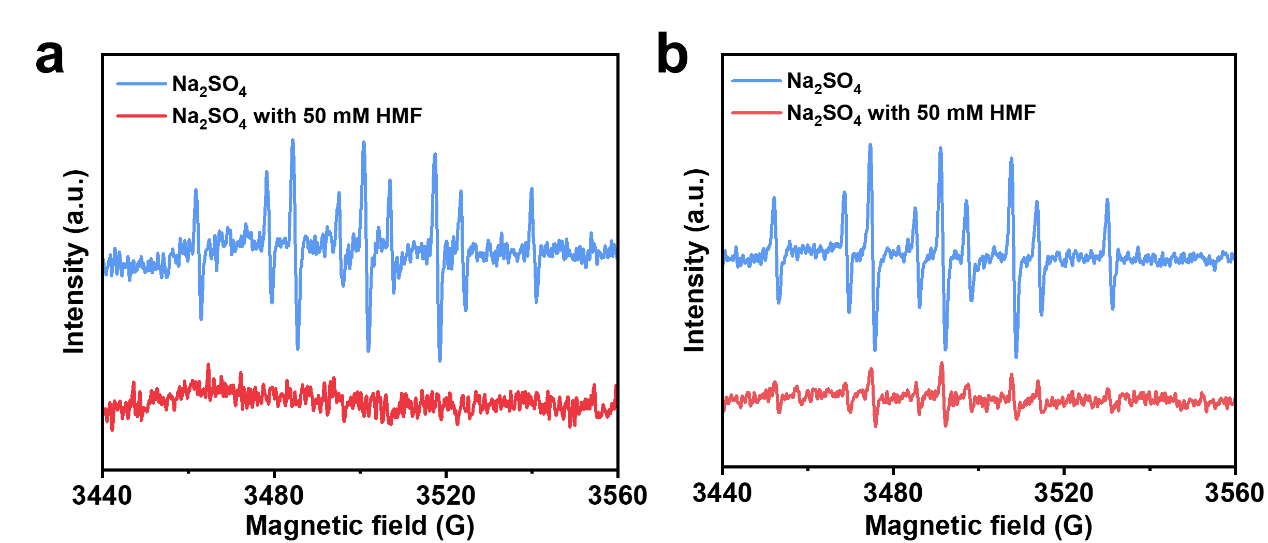


Figure S36. Quasi-in situ EPR trapping for hydrogen radical over Cu_2_O (a) and Cu_2_O/Co_3_O_4_ (b) with the addition of 50 mM HMF.

**
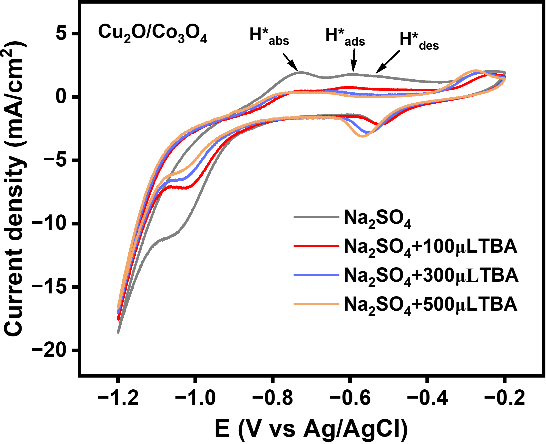
**

Figure S37. CV curves of Cu_2_O/Co_3_O_4_ in 0.1 M Na_2_SO_4_ with various concentrations of t-Butanol at a scan rate of 10 mV·s^-1^.


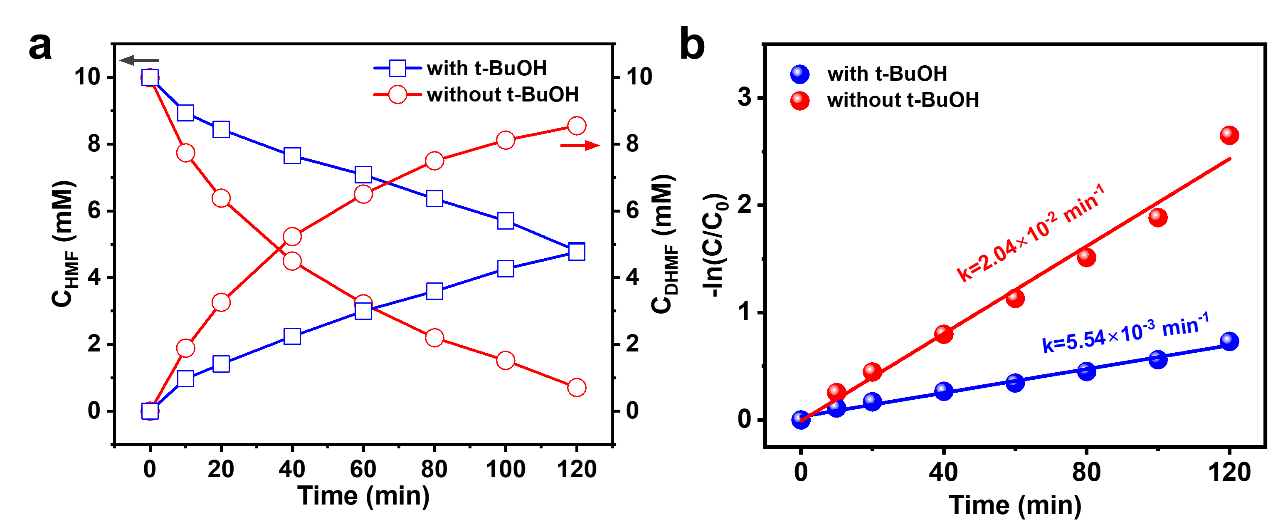


Figure S38. (a) HMF and DHMF conversion of Cu_2_O/Co_3_O_4_ with and without t-BuOH addition at -0.6 V vs. RHE. (b) Kinetic fitting curves for the reduction of 5-HMF to DHMF with and without t-BuOH addition at -0.6 V vs. RHE.


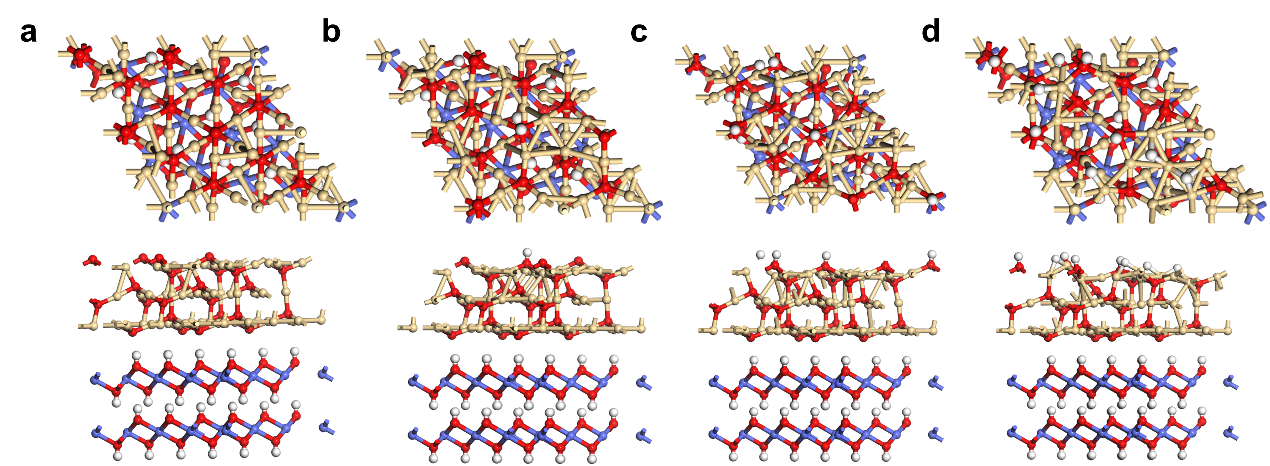


Figure S39. Optimized configurations on Cu_2_O/Co_3_O_4_ surface under 0 *H nm^-2^ (a), 1 *H nm^-2^ (b), 4 *H nm^-2^ (c), and 8 *H nm^-2^ (d) coverage conditions.


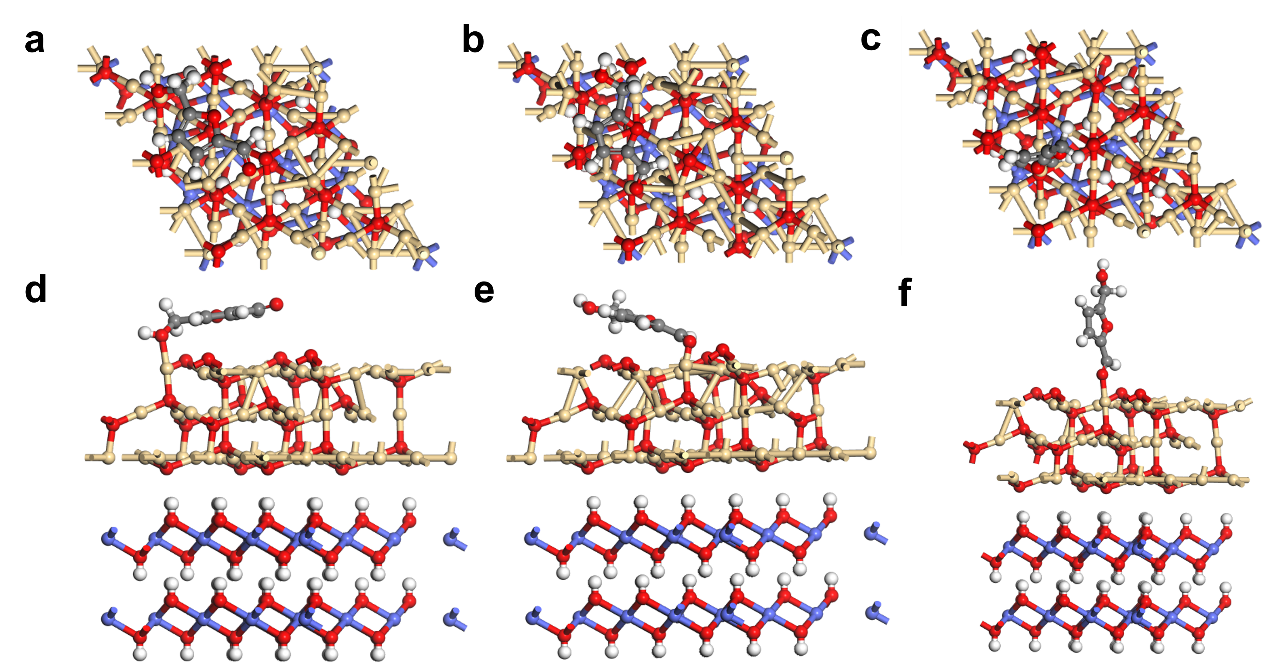


Figure S40. Optimized adsorption configurations of HMF on Cu_2_O/Co_3_O_4_ Surface: (a, d) flat-Lying, (b, e) tilted, and (c, f) vertical Orientations.


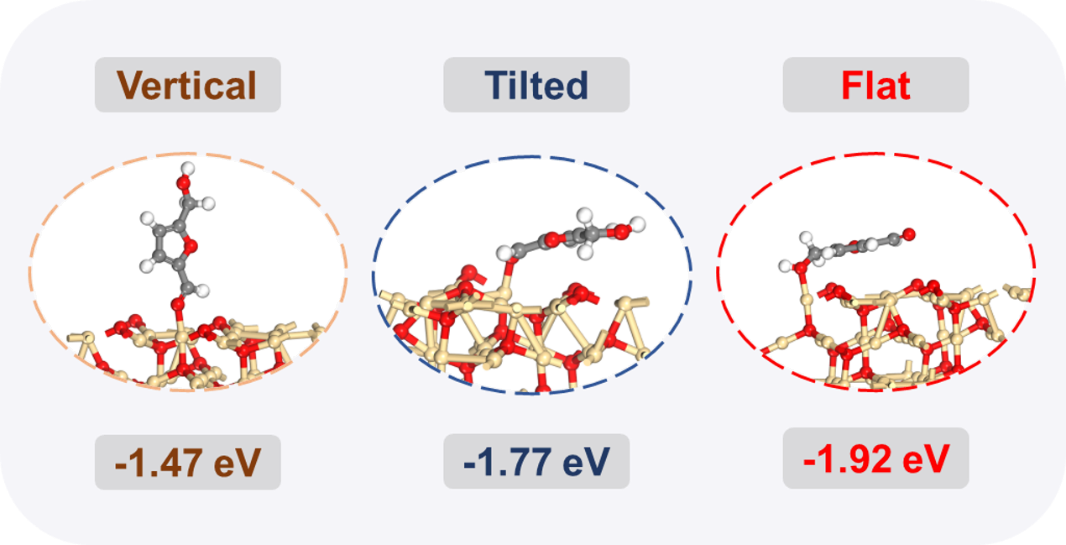


Figure S41. The optimized geometric structures and the corresponding adsorption energies of 5-HMF adsorption on Cu_2_O/Co_3_O_4_.


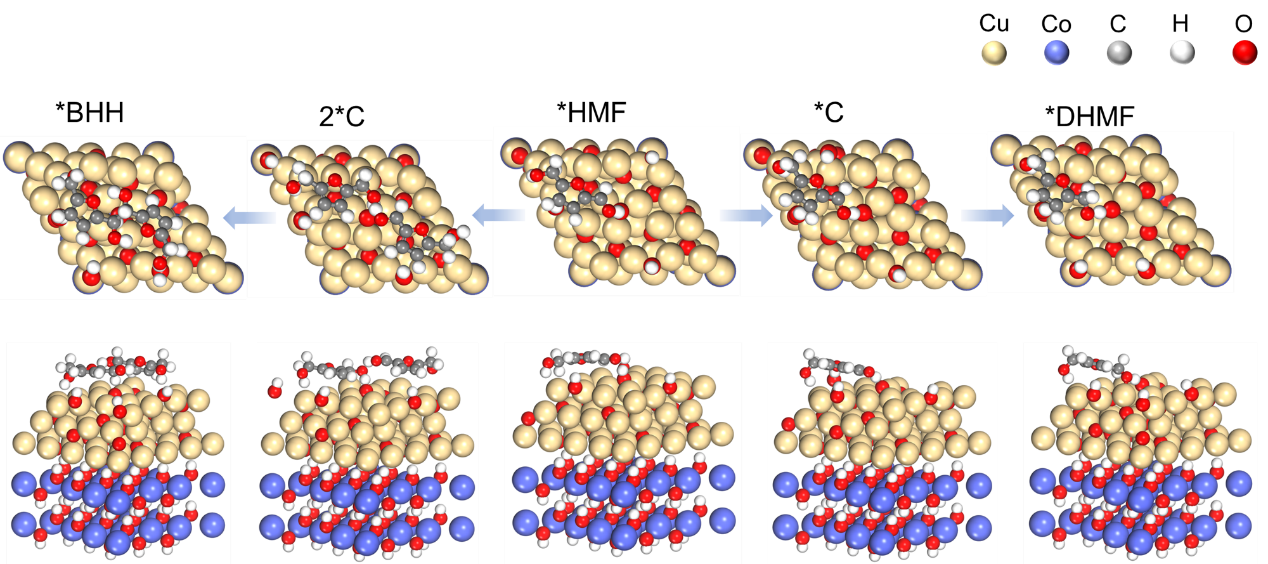


Figure S42. Schematic illustration of the formation of DHMF and BHH during the HMFRR process on Cu_2_O/Co_3_O_4_ under 4 *H coverage.

Figure S43. The COHP analysis of O-H in Cu_2_O and Cu_2_O/Co_3_O_4_.

Supplementary tables

Table S1. Post-Cycle Electrolyte Metal Leaching of Cu_2_O/Co_3_O_4._

| Ion species | Ion concentration （mg/L） | Leaching ratio (%) |
| --- | --- | --- |
| Cu | 0.427 | 0.15 |
| Co | 0.129 | 0.046 |

Table S2. Comparative summary of the reported HMFRR performance

| Catalyst | Electrolyte | pH | Potential  (V *vs*. RHE) | 5-HMF Conversion (%) | DHMF Selectivity (%) | Faradaic efficiency (%) | Ref. |
| --- | --- | --- | --- | --- | --- | --- | --- |
| PdCu | 0.1 M PBS | - | -0.47 | 89 | 99 | - | ^[8]^ |
| Pt_1%_-Cu | 0.1 M PBS | 7 | -0.3 | - | 95.1 | 91.2 | ^[9]^ |
| CoCuMW/CF | 0.5 M PBS | 7 | -0.5 | 95.7 | 89.2 | - | ^[10]^ |
| CuPS_3_ | 0.1 M PBS | 9.2 | -0.7 | - | - | 91.3±2.3 | ^[11]^ |
| RhCu | 0.1 M PBS | 7 | -50 mA cm^-2^ | 71.9 | 99.7 | 72.4 | ^[12]^ |
| Ru_1_Cu | 0.5 M PBS | 7 | -0.3 | 87.3 | 97.5 | 85.6 | ^[13]^ |
| Ag/C | 0.5 M sodium borate buffer | 9.2 | -0.56 | 42 | 90 | 95 | ^[14]^ |
| AgCu | 0.5 M sodium borate buffer | 9.2 | -0.56 | 53 | 87 | 95 | ^[15]^ |
| OD-Ag | 0.5 M sodium borate buffer | 9.2 | -0.56 | 37 | 91.7 | 56.2 | ^[16]^ |
| 15%-Cu/NC_900_ | 1 M KOH | 13.6 | -0.25 | 81 | 94 | - | ^[17]^ |
| Co_3_O_4_ | 0.1 M PBS | - | -0.47 | 96 | 83.3 | 26.3 | ^[18]^ |
| Ag@CuF | 1 M PBS | 7.2-7.4 | -0.66 | 100 | 93.2 | 43 | ^[19]^ |
| Pd Nps/XC-72 | 1 M PBS | 6.8 | -0.75 | 100 | 89.63 | 52 | ^[20]^ |
| FeO_x_/Fe-IF | 0.1 M Na_2_SO_4_ | 7 | -0.54 | 85.2 | 100 | 39.0 | ^[21]^ |
| Cu_2_O/Co_3_O_4_ | 0.1 M Na_2_SO_4_ | 7 | -0.60 | 97% | 97% | - | This  work |
|  | 0.1 M Na_2_SO_4_ | 7 | -0.55 | 88.3% | 98.2 | 54.5 |  |
|  | 0.1 M Na_2_SO_4_+5mM 15-C-5 | 7 | -0.55 | 78.6% | 97.7% | 62.5% |  |

Table S3. Time-Resolved Evolution of Co²⁺ Peak Area Ratios in XPS

| Reaction time (min) | Peak area of Co^2+^ 2p3/2 | Peak area of Co^3+^ 2p3/2 | Ratio of Co^2+^  (%) |
| --- | --- | --- | --- |
| 0 | 9031.3 | 12721.1 | 41.5 |
| 30 | 5350.3 | 4923.3 | 52.1 |
| 60 | 8012.6 | 5176.8 | 60.8 |
| 90 | 9932.3 | 3635.9 | 73.2 |
| 120 | 17712.9 | 0 | 100 |

Table S4. Optimum fit parameters for the electrochemical impedance spectra of Cu_2_O/Co_3_O_4_ in 0.1 M Na_2_SO_4_ with and without HMF

| Electrolyte | Potential  (V vs. RHE) | R_s_  (Ω) | R_1_  (Ω) | CPE1  (Fs ^n-1^) | n | R_2_  (Ω) | C_Ф_  (F) |
| --- | --- | --- | --- | --- | --- | --- | --- |
| 0.1 M Na_2_SO_4_ | -0.40 | 9.087 | 3.149 | 0.02679 | 0.8027 | 39.18 | 0.008589 |
|  | -0.45 | 9.038 | 2.728 | 0.02453 | 0.8111 | 24.63 | 0.09221 |
|  | -0.50 | 9.274 | 2.688 | 0.01559 | 0.8283 | 10.56 | 0.006010 |
|  | -0.55 | 9.062 | 1.146 | 0.02332 | 0.7793 | 10.04 | 0.009483 |
|  | -0.60 | 9.188 | 1.184 | 0.01807 | 0.8029 | 6.322 | 0.006955 |
|  | -0.65 | 9.112 | 0.8606 | 0.001708 | 0.8300 | 4.389 | 0.01108 |
|  | -0.70 | 9.156 | 0.8001 | 0.01354 | 0.8463 | 3.013 | 0.01000 |
| 0.1 M Na_2_SO_4_  +10 mM HMF | -0.40 | 10.39 | 2.613 | 0.02303 | 0.7513 | 33.36 | 0.004351 |
|  | -0.45 | 10.34 | 2.397 | 0.01906 | 0.7726 | 24.85 | 0.005574 |
|  | -0.50 | 10.31 | 1.691 | 0.01494 | 0.8003 | 17.04 | 0.006835 |
|  | -0.55 | 10.30 | 1.444 | 0.01360 | 0.8222 | 11.38 | 0.006950 |
|  | -0.60 | 10.20 | 1.064 | 0.01118 | 0.8520 | 8.285 | 0.007649 |
|  | -0.65 | 10.21 | 1.009 | 0.008483 | 0.8956 | 5.597 | 0.009235 |
|  | -0.70 | 10.21 | 0.9069 | 0.008172 | 0.9019 | 4.160 | 0.009291 |

Table S5. Optimum fit parameters for the electrochemical impedance spectra of Cu_2_O in 0.1 M Na_2_SO_4_ with and without HMF

| Electrolyte | Potential  (V vs. RHE) | R_s_  (Ω) | R_1_  (Ω) | CPE1  (Fs ^n-1^) | n | R_2_  (Ω) | C_Ф_  (F) |
| --- | --- | --- | --- | --- | --- | --- | --- |
| 0.1 M Na_2_SO_4_ | -0.40 | 11.55 | 3.948 | 0.0009154 | 0.8195 | 50.92 | 0.0001012 |
|  | -0.45 | 11.41 | 2.617 | 0.001174 | 0.7840 | 45.10 | 0.0001450 |
|  | -0.50 | 11.42 | 2.509 | 0.001149 | 0.7901 | 24.35 | 0.0001837 |
|  | -0.55 | 11.42 | 2.515 | 0.0009359 | 0.8167 | 11.58 | 0.0002451 |
|  | -0.60 | 11.31 | 2.072 | 0.0007744 | 0.8545 | 6.079 | 0.0003468 |
|  | -0.65 | 11.24 | 1.598 | 0.0009404 | 0.8209 | 4.134 | 0.0004850 |
|  | -0.70 | 11.21 | 1.417 | 0.001279 | 0.7919 | 2.860 | 0.0005672 |
| 0.1 M Na_2_SO_4_  +10 mM HMF | -0.40 | 11.92 | 2.104 | 0.002158 | 0.7248 | 44.52 | 0.0003098 |
|  | -0.45 | 12.08 | 2.309 | 0.002827 | 0.7022 | 40.83 | 0.0004837 |
|  | -0.50 | 12.03 | 2.564 | 0.002453 | 0.7350 | 27.27 | 0.0005220 |
|  | -0.55 | 11.98 | 2.376 | 0.002136 | 0.7491 | 16.81 | 0.0006650 |
|  | -0.60 | 12.03 | 1.984 | 0.001988 | 0.7572 | 9.838 | 0.0007717 |
|  | -0.65 | 12.05 | 1.599 | 0.002108 | 0.7521 | 6.307 | 0.0008216 |
|  | -0.70 | 11.92 | 1.576 | 0.002025 | 0.7606 | 4.508 | 0.0008557 |

**References**

[1] K. Ji, M. Xu, S. M. Xu, Y. Wang, R. Ge, X. Hu, X. Sun, H. Duan, Electrocatalytic Hydrogenation of 5-Hydroxymethylfurfural Promoted by a Ru_1_Cu Single-Atom Alloy Catalyst, Angew. Chem. Int. Ed. **2022**, 61, e202209849.

[2] J. Anibal, B. Xu, Electroreductive C–C Coupling of Furfural and Benzaldehyde on Cu and Pb Surfaces, ACS Catal. **2020**, 10, 11643-11653.

[3] Y. Z. Ji, Z.; Duan, A.; Jiang, G.; Liu, J, Comparative Study on the Formation and Reduction of Bulk and Al_2_O_3_-Supported Cobalt Oxides by H_2_-TPR Technique, J. Phys. Chem. C **2009**, 113 (17).

[4] W. Zhu, X. Chen, C. Li, Z. Liu, C. Liang, Manipulating morphology and surface engineering of spinel cobalt oxides to attain high catalytic performance for propane oxidation, Journal of Catalysis **2021**, 396, 179-191.

[5] Q. Hua, K. Chen, S. Chang, H. Bao, Y. Ma, Z. Jiang, W. Huang, Reduction of Cu_2_O nanocrystals: reactant-dependent influence of capping ligands and coupling between adjacent crystal planes, RSC Advances. **2011**, 1.

[6] H. Z. Bao, W.; Shang, D.; Hua, Q.; Ma, Y.; Jiang, Z.; Yang, J.; Huang, W., Shape Dependent Reducibility of Cuprous Oxide Nanocrystals, J. Phys. Chem. C. **2010**, 114 (14), 6676-6680.

[7] B. Wei, N. Yang, F. Pang, J. Ge, Cu_2_O–CuO Hollow Nanospheres as a Heterogeneous Catalyst for Synergetic Oxidation of CO, The Journal of Physical Chemistry C. **2018**, 122, 19524-19531.

[8] W. Z. Xu Yue, Shuangyin Wang and Yuqin Zou, Selective electrocatalytic hydrogenation of 5-hydroxymethyl-furfural to 2,5-dihydroxymethylfuran on bimetallic PdCu alloy, Chin. J. Struct. Chem. **2022**, 41, 2205063-2205069.

[9] Y. Gao, C. Tang, Y. Zheng, Manipulating adsorbed hydrogen for enhanced HMF electrocatalytic hydrogenation, J. Energy Chem. **2025**, 105, 439-445.

[10] B. Zhu, J. Yang, Q. Wang, X. Yu, S. Fan, W. Xie, J. Zhang, C. Chen, Corrosion-induced CoCu microwire arrays for efficient electroreduction of 5-hydroxymethylfurfural, Chem. Commun. **2025**, 101259.

[11] M. G. Sendeku, K. Harrath, F. T. Dajan, B. Wu, S. Hussain, N. Gao, X. Zhan, Y. Yang, Z. Wang, C. Chen, W. Liu, F. Wang, H. Duan, X. Sun, Deciphering in-situ surface reconstruction in two-dimensional CdPS_3_ nanosheets for efficient biomass hydrogenation, Nat. Commun. **2024**, 15, 5174.

[12] W. Zhang, W. Ge, Y. Qi, X. Sheng, H. Jiang, C. Li, Surfactant directionally assembled at the electrode-electrolyte interface for facilitating electrocatalytic aldehyde hydrogenation, Angew. Chem. Int. Ed. **2024**, 63, e202407121.

[13] K. Ji, M. Xu, S.-M. Xu, Y. Wang, R. Ge, X. Hu, X. Sun, H. Duan, Electrocatalytic hydrogenation of 5-hydroxymethylfurfural promoted by a Ru_1_Cu single-atom alloy catalyst, Angew. Chem. Int. Ed. **2022**, 61, e202209849.

[14] X. H. Chadderdon, D. J. Chadderdon, T. Pfennig, B. H. Shanks, W. Li, Paired electrocatalytic hydrogenation and oxidation of 5-(hydroxymethyl)furfural for efficient production of biomass-derived monomers, Green Chem. **2019**, 21, 6210-6219.

[15] L. Zhang, F. Zhang, F. C. Michel Jr, A. C. Co, Efficient electrochemical hydrogenation of 5-hydroxymethylfurfural to 2,5-bis(hydroxymethyl)furan on Ag-displaced nanotextured Cu catalysts, ChemElectroChem **2019**, 6, 4739-4749.

[16] H. Liu, T.-H. Lee, Y. Chen, E. W. Cochran, W. Li, Paired electrolysis of 5-(hydroxymethyl)furfural in flow cells with a high-performance oxide-derived silver cathode, Green Chem. **2021**, 23, 5056-5063.

[17] W. Xu, C. Yu, J. Chen, Z. Liu, Electrochemical hydrogenation of biomass-based furfural in aqueous media by Cu catalyst supported on N-doped hierarchically porous carbon, Appl. Catal. B **2022**, 305, 121062.

[18] X.-Q. Pan, X.-Y. Zhang, G.-X. Huang, S.-C. Mei, J.-W. Huang, J.-J. Chen, W.-J. Liu, H.-Q. Yu, Promoting electrocatalytic hydrogenation of 5-hydroxymethylfurfural using buffer electrolytes as proton-donating motifs: Theoretical predictions and experimental validations, Appl. Catal. B **2023**, 323, 122191.

[19] Z. Dai, X. Liu, N. Liu, Y. Zhang, X. Zhao, Upgrading biomass derived furan aldehydes by coupled electrochemical conversion over silver-based electrocatalysts, Chem. Eng. J. **2024**, 488, 151001.

[20] Y. Wu, Y. Jiang, W. Chen, X. Yue, C.-L. Dong, M. Qiu, T. T. T. Nga, M. Yang, Z. Xia, C. Xie, L. Xu, R. Wang, S. Wang, Y. Zou, Selective electroreduction of 5-hydroxymethylfurfural to dimethylfuran in neutral electrolytes via hydrogen spillover and adsorption configuration adjustment, Adv. Mater. **2024**, 36, 2307799.

[21] X. Y. Zhang, S. S. Yu, X. X. Shu, J. R. Wang, J. J. Chen, H. Q. Yu, Balancing the Activity and Stability of Iron‐Based Catalysts via Mixed Oxide Species Formation for Electrocatalytic Biomass Hydrogenation, Adv. Funct. Mater. preprint **2025**, DOI: 10.1002/adfm.202504594.
